# *NAUNEHAL;* Integrated immunization and MNCH interventions: A quasi-experimental study–Protocol

**DOI:** 10.1371/journal.pone.0287722

**Published:** 2023-06-29

**Authors:** Anushka Ataullahjan, Amira Khan, Muhammad Islam, Rehman Tahir, Saeed Anwar, Imran Ahmed, Ahmed Nauman, Zulfiqar A. Bhutta

**Affiliations:** 1 Centre for Global Child Health, Hospital for Sick Children, Toronto, Ontario, Canada; 2 Trust for Vaccines and Immunization, Karachi, Pakistan; 3 Prime Foundation, Peshawar Medical College, Peshawar, Pakistan; 4 Centre for Excellence in Women and Child Health, Aga Khan University, Karachi, Pakistan; Marie Stopes International, PAKISTAN

## Abstract

**Introduction:**

Great improvements in the health of newborns, children, and women in Pakistan are needed. A large body of literature has demonstrated that the majority of maternal, newborn, and child deaths are preventable with essential health strategies including immunization, nutrition interventions, and child health interventions. Despite the importance of these interventions for the health of women and children, access to services continues to be a barrier. Furthermore, demand for services also contributes to low coverage of essential health interventions. Given the emerging threat of COVID-19 coupled with already weak maternal and child health, delivering effective and feasible nutrition and immunization services to communities, and increasing demand and uptake of services is a pressing and important need.

**Methods and analysis:**

This quasi-experimental study aims to improve health service delivery and increase uptake. The study included four main intervention strategies including community mobilization, mobile health teams offering MNCH and immunization services, engagement of the private sector, and testing of a comprehensive health, nutrition, growth, and immunization app, Sehat Nishani, for a period of 12 months. The target group of the project were women of reproductive age (15–49 years) and children under-five. The project was implemented in three union councils (UCs) in Pakistan including Kharotabad-1(Quetta District, Balochistan), Bhana Mari (Peshawar District, Khyber Pakhtunkhwa) and Bakhmal Ahmedzai (Lakki Marwat district, Khyber Pakhtunkhwa). Propensity score matching based on size, location, health facilities, and key health indicators of UC was conducted to identify three matched UCs. A household baseline, midline, endline and close-out assessment will be conducted for evaluating coverage of interventions as well as the knowledge, attitude, and practices of the community in the MNCH and COVID-19 context. Descriptive and inferential statistics will be used to test hypotheses. As well, a detailed cost-effectiveness analysis will be conducted to generate costing data for these interventions to effectively inform policymakers and stakeholder on feasibility of the model.

**Trial registration:** NCT05135637.

## Introduction

The health of newborns, children, and adolescents in Pakistan is an area where progress and improvements are greatly needed [1]. Though slow gains have been made in improving maternal, newborn and child health (MNCH) indicators, the country’s under-five mortality rate still stands at 74 deaths per 1000 live births and the maternal mortality rate at 186 deaths per 100,000 livebirths [2, 3]. Alarmingly, Pakistan has the highest neonatal mortality rate globally at 42 neonatal deaths per 1000 live births [2]. A large body of literature has demonstrated that the majority of these maternal, newborn, and child deaths are preventable with essential health interventions including immunizations, nutrition interventions, and child health interventions [4]. However, access to care can be a barrier due to a poorly functioning health system limiting the provision of services [4]. Rural-urban and socioeconomic disparities and consequent health inequities also contribute to vulnerable populations experiencing worsened health indicators. Limited demand for vaccines due to sociocultural beliefs influences the demand for services, and vaccine hesitancy continues to limit vaccine uptake.

COVID-19 and related mitigation strategies to decrease its spread presented health systems globally with a daunting challenge, including the Pakistani health system. The disruption of health service delivery and reduced care seeking has increased the risk of disease and malnutrition in vulnerable populations. From April to July 2020, vaccination drives and polio supplementary immunization activities were halted, with routine vaccination rates falling owing to fear in the communities of visiting health facilities [5, 6]. As the Government of Pakistan works to strengthen the existing health systems in the context of conflict and COVID-19, it is critical that an integrated approach is taken which focuses on effective delivery platforms for providing health and immunization services where they are most required. Research models indicate that scaling up interventions in Pakistan by using appropriate delivery platforms to reach rural and vulnerable populations, via community-based strategies, could reduce maternal, newborn and child deaths by nearly 60% [7].

Given the impact of the private sector on the health system, engaging private health providers will also be an important part of any integrated strategy to improve maternal and child health [8]. In Pakistan, it is estimated that the private sector accounts for nearly three-quarters of total health services delivered [9]. Private health providers’ role in ensuring access to maternal and child health services and immunization is substantial. In these challenging contexts, private health providers can increase the availability of essential health services to high-risk communities who have limited options and poor coverage of interventions. Despite their potential importance, there has been limited research on the most effective ways to engage with the private sector.

Further to the barriers to delivering services, limited demand for services also underlies low health intervention coverage. Increasing uptake of vaccine services has proved to be a large barrier in Pakistan. Polio vaccination incites specific fears related to potential side effects, suspicions about the motives of the program, and the religious permissibility of the vaccine [10]. Addressing these concerns has been an issue for polio eradication efforts in Pakistan. Research on polio in Pakistan has demonstrated that the refusal of services is, in fact, not solely a reflection of an aversion to the service but also a lack of trust in the authorities providing services [11]. Early community engagement, particularly the engagement of elders and religious leaders can act as mechanism through which to dispel any fears or concerns related to the services provided. This is of particular importance as the literature has demonstrated exclusion from systems of power without the opportunity to ask questions can contribute to misconceptions and conspiracy theories about health services [12, 13].

Given the impact of COVID-19, coupled with already weak maternal and child health status in Pakistan, delivering effective and sustainable MNCH, nutrition and immunization services to in-need communities throughout the country is a pressing priority. Increasing efficiency through integration and increasing demand for services through improving community awareness and care seeking behaviours is also imperative to improve maternal and child health. Recognizing these challenges and opportunities, *Naunehal* aims to integrate MNCH and immunization strategies through community mobilization and private sector engagement, to create a replicable and cost-effective model that can potentially be adopted by the government for the long-term sustainability of the interventions. In the context of COVID-19, these steps have even more relevance to rebuilding primary care and immunization systems. The project also tests the use of a health, nutrition and immunization app, *Sehat Nishani* which creates an integrated interface between immunization registry, growth records and household data, to help track coverage better.

## Materials and methods

### Study objectives

- Improve coverage of MNCH practices and services, particularly in the COVID-19 context including routine childhood immunization, improved breastfeeding practices, handwashing promotion, best possible sanitary practices, and optimal diarrhea management.
- Enhance community engagement and knowledge to improve care seeking and health behaviours.
- Create a replicable model of private health sector engagement that can be adopted by the
- government for long-term sustainability of the interventions.
- Examine the feasibility of the intervention by assessing the cost-effectiveness of this model of delivery.
- Develop and pilot test a cell phone app that integrates immunization registry, birth record, child health and growth information and household data.

### Study setting

*Naunehal* was implemented in three union councils (UCs) of Pakistan, Kharotabad 1(Quetta District, Balochistan Province) and Bhana Mari (Peshawar District, Khyber Pakhtunkhwa (KP) Province) are classified as super high-risk UCs (SHRUCs) for polio, while UC Bakhmal Ahmedzai in district Lakki Marwat, KP, is an area which has experienced polio outbreaks recently (Appendix A in S1 File). In addition to high polio virus transmission, the three UCs have sub-optimal immunization coverage, maternal and under-five health, and nutrition indicators and were thus chosen for the Naunehal project.

### Study design

The project adopts a quasi-experimental prepost study design to assess the impact of the proposed interventions. The three UCs are the intervention sites and for each of these target UCs, a separate, matched control UC of comparable size, population, location and coverage indicators was identified, with propensity score matching and in consultation with local partners. The matched UC was always in the same district but never bordering the target UC. Baseline, midline endline, and close-out surveys will be conducted using the 30x7 techniques. Thirty clusters demarcated by the government’s polio program from each UC are randomly selected and 15 households with children under-five selected from each cluster. Thus, to achieve an optimal sample size, 450 households are surveyed from each of the intervention and control UCs (Table 1). The surveys focus on household characteristics, immunization coverage, health & care seeking behaviours, especially in the COVID context, and water, sanitation, and hygiene (WASH) practices.

**Table 1:** Sample size for household survey by UC.

Sr No.	District	Sample Size (Households)
1.	UC in Quetta	450
2.	UC in Peshawar	450
3.	UC in Lakki Marwat	450
4.	Matched control UC in Quetta	450
5.	Matched control UC in Peshawar	450
6.	Matched control UC in Lakki Marwat	450
Total		2,700

#### Intervention strategies

*Naunehal* aims to improve health service delivery and enhance uptake, by focusing on four main intervention strategies implemented in all three target UCs; community mobilization to enhance awareness and demand, mobile health teams offering MNCH and immunization services, engagement of private sector to increase immunization coverage and testing of a comprehensive health, nutrition, growth, and immunization app, *Sehat Nishani*. The target group of the project include women of reproductive age (WRA) (15–49 years) and children under-five residing in the UCs.

**Community engagement and mobilization** includes dissemination of comprehensive messaging on promotion of routine childhood immunization, optimal infant and young child feeding (IYCF) practices, handwashing, best possible sanitary practices, COVID-19 prevention, optimal diarrhea management, and appropriate care seeking for both pregnant women and young children. The messages were communicated via health promotion materials, health awareness sessions, and mosque announcements. Announcements especially encouraged the community for routine childhood immunization and to utilize *Naunehal* mobile health services and visit private practitioners’ clinics or government facilities for immunization. An integral step of the community engagement process was to map and consult with opinion leaders and influential community members, to introduce the project and gain their support for the activities.

**Mobile health services (MHS)** were deployed in each of the three pilot UCs, with *one team per UC* in the pilot phase. Each mobile team had *a female physician*, *a female vaccinator*, *and a facilitator* and visited pre-determined areas of the UC daily, for 06 days per week for 12 months. The locations kept rotating, with an effort to focus on hard-to-reach areas where access to and coverage of health facilities is limited. The team ensured that all COVID-19 -prevention recommendations of the government are followed.

The MHS provided basic health services, immunization, and health and IYCF counselling targeted towards children under-five and WRAs. The immunization services provided by the team are limited to those already approved and recommended by the Pakistan government (Appendix B in S1 File). The physician treated the young children for common childhood illnesses such as diarrhea and acute respiratory infections. Those children or WRAs requiring referral or inpatient care were referred to the nearest health facility. Consent was taken from caregivers of under-five children and participants so that details on services delivered can be shared and utilized in study analysis. Participants received services regardless of whether consent for data collection was given or not. Personal identifiers will be removed from data.

The project did not anticipate any major adverse event as routine vaccines are widely used safely. However, guidance was provided to the team physician on steps to follow in case of an adverse event, including providing emergency medication, stabilization, subsequent referral to a health facility if needed, following up on the child, and directive to return to camp after 24–48 hours for check-up. Serious and/or unexpected adverse events were to be evaluated and reported to Aga Khan University (AKU) and the SickKids Centre for Global Child Health. The information on all events was collected, assessed, and reported on Case Report Forms (CRFs) through routine reporting. If a subject suffered any injury while receiving care, he/she received free of cost, appropriate care as required and the organization would bear the cost of this care.

**Private Sector Engagement** is an innovative strategy where Naunehal aimed to create a replicable model of private sector engagement that could be adopted by the government for long-term sustainability. Private sector healthcare providers (HCPs) in the target UCs were taken on board to increase coverage of routine immunization and improve the quality of maternal and child health services. HCPs offered age-appropriate routine vaccination, including missed doses, to all under-five children visiting their clinics.

Identification of HCPs occured during the line listing of the baseline survey, and HCPs with a medical degree who were willing and whose clientele included under-five children were selected. The engaged HCPs were trained in collaboration with the District Health Office and the Expanded Programme on Immunization (EPI). Training primarily focused on immunization of neonates and young children as per the schedule and methods endorsed by EPI. Safety protocols, cold chain maintenance and vaccination recordkeeping were an integral component of the training program. As well, the HCPs were trained on use of Naunehal health promotion materials and counselling.

Project team liaised with EPI to arrange vaccines for the private practitioners and supply them on a regular, as-needed basis. The arrangement ensured that vaccines were ready to be picked up or delivered on a pre-determined day from the EPI office with cold-chain maintenance preparations to the practitioners’ clinic. These included BCG, OPV, IPV, Pentavalent, Pneumococcal, Measles and Rotavirus vaccines.

An incentive for the practitioners was to receive much promotion and exposure within the UC, during community mobilization and public announcements. As well, it was an opportunity for them to build linkages with the community and with government partners. Non-monetary incentives in the form of equipment may be offered to practitioners who administer a larger number of vaccines.

***Sehat Nishani*,** a digital health mobile application was launched in the three intervention UCs to assess the feasibility of adopting a birth, health, and immunization app to create a vaccination database and capture information on birth, birth weight, nutrition and growth records essential for follow up of the cohort. The app was developed in English and Urdu at AKU’s Technology Innovation Support Centre, specifically for Naunehal and the Urdu version was piloted during the project. Lady health workers (LHWs), lady health visitors (LHVs) in government health facilities, EPI vaccinators and partnering HCPs were engaged to use the *Sehat Nishani* app on their existing Android phones.

The health workers received the necessary orientation and training to use the app and received technical support when needed from the project team. Health workers had app access via a unique login ID and password and thus, the app was not publicly accessible. Data from a single family was linked via a unique Family ID also accessible via an assigned QR code. While LHWs and HCPs entered both vaccination and health data for under-two children and WRAs, the vaccinators only entered vaccination data. Health data for children included birth history, nutritional history, growth record, and an illness record. For WRAs, in addition to the nutritional and illness history, the app was designed to record details on antenatal care, delivery and postnatal care. Caregivers were able to log in to the app with their unique ID number and see their child’s record without making any changes or entering any information. All data in the app will be encrypted to ensure patient and household information stored is secure.

App records are stored in a cloud-based central database managed by AKU, where child health and nutrition data, vaccinator performance, vaccination rates, children overdue for vaccination, and under-immunized areas can be identified.

### Monitoring and impact evaluation

*Naunehal* has undergone rigorous monitoring and evaluation. As mentioned, household surveys are to be conducted at baseline, midline, at the end of the project to assess the impact of interventions and one year after to examine sustained effect.

The Mother & Child Care Trust (MCCT), an independent non-governmental organization based in Karachi, Pakistan, working on maternal and child health research projects, conducted an independent monitoring and evaluation of the project. MCCT has tracked the impact of the project using a range of indicators including the number of births registered, birth dose of OPV and BCG received, receipt rates of pneumococcal conjugate vaccine (PCV) and measles vaccine, immunization rates for all vaccines, and zero dose cases.

#### Household surveys

A household baseline, midline, endline and close-out assessment will be conducted for evaluating coverage of interventions as well as the knowledge, attitude, and practices of the community in the MNCH and COVID-19 context.

The inclusion criteria for a household includes consent from the family, at least one child less than 5 years of age in the household, and that the family has been living in that high-risk UC for at least 6 months (including the child in the household) prior to the day of the interview. However, the household is eligible even if the only child in household was born in the last six months. For close-out survey, the families should have been living in the UC for at least one year. Any household where a parent or caregiver is not available to answer questions related to the child’s health and immunization, will be *excluded*. Data collected will be processed and analyzed at AKU, Karachi.

A consent form outlining the objectives of the study, how privacy concerns will be handled, and how the data will be used is shared with the participants. They are given as much time as needed to review the consent form and ask any additional questions. If they feel comfortable, they can sign the form and the survey commences. If not, they are thanked for their time and not included in the survey.

The surveys will help to develop a comprehensive picture of population distribution, health intervention, care seeking and immunization coverage for children under-5 years. The baseline (Appendix C in S1 File) serves as the basis for targeting and tracking progress. Given the enormous impact that COVID-19-related mitigation strategies have had on routine health services such as antenatal care, childbirth services, immunizations and care seeking, the baseline assessment also assessed bottlenecks and helped tailor possible approaches to address these issues. The endline survey (Appendix D in S1 File) was conducted immediately after conclusion of intervention while the close-out survey will be conducted, a year after the endline to explore presence of sustained effects and persistent benefit. Implementation was conducted from 2021 to 2022, while the endline survey was conducted in 2022. The close-out survey will be conducted in 2023, while the cost-effectiveness data will also be compiled in the same year. The close-out survey, to be conducted one year after completion of intervention, will aim to assess retention, long-term benefits, and sustainability of the intervention especially with a focus on immunization coverage, IYCF practices, and care seeking behaviours.

#### Cost-effectiveness analysis

The cost-effectiveness assessment will focus on the complete package and then separate, principal components including health, immunization, and nutrition interventions. The costing methodology will be activity-based, with distinct implementation costs. The costing data will include community engagement, mobile health services, and private practitioner engagement. Costs incurred, incremental costs, costs averted, and predicted and identified change in health, immunization and nutrition outcomes will inform the analysis.

### Analysis

All data is captured electronically using customised applications running on android based handheld devices. Data is hosted at the Aga Khan University data center. Demographic, clinical, knowledge, attitude & practices (KAP), and MNCH related indicators data will be compiled and analyzed using STATA -version 17.0 [14].

Descriptive and inferential statistics will be used to characterize the study sample and test hypotheses. Descriptive results for all continuous variables (e.g., age) will be presented as mean ± standard deviation or median with inter-quartile range, while numbers (percentage) will be reported for all categorical variables.

To assess the independent effect of intervention, propensity score matching was employed to identify similar UCs based on geographical location of UC, population density, size of under-five population, number of health facilities and health workers, and immunization coverage. Through this process, three matched UCs are also surveyed. Among the predictors, exact matching has been enforced to achieve the balance for all predictors between the intervention and non-intervention groups.

We will calculate overall and region-specific health coverage at baseline, midline, endline and at close-out for both intervention and control group. The mean and percentage point difference in coverage between baseline and endline, and baseline and close-out will be estimated using a mixed effects linear regression model. Effect estimates will be reported with 95% confidence intervals. The clustered nature of the data will be accounted for by including each cluster as a random effect. Estimates will be adjusted for survey design and sampling weights by treating each UC as strata and clusters as primary sampling units.

Costing data for the *Naunehal* program will be collected based on a standardized template developed by J-PAL [15]. It will be explicitly mentioned what costs are being recorded including project administration, training, implementation, goods purchased, and monitoring. The costs will be measured with the consideration that *Naunehal* is a pilot project and scaling up the project may change actual costs. Base Year and Year of Analysis will be accounted for including inflation costs and exchange rates. The cost-effectiveness will be assessed on basis of number of beneficiaries reached and the program effect on key indicators, followed by determining the cost-effectiveness ratio.

### Ethics and dissemination

Ethics approval was obtained from the SickKids Research Ethics Board (#1000072563), and National Bioethics Committee (#NBC-561) based in Pakistan. Informed consent is obtained from all survey respondents i.e. the parents or guardians of under-five children. Importantly, No Objection Certificates (NOCs) were secured from authorities in Balochistan and KP. Community buy-in and support for the project is pivotal to its success. Meetings with community elders and leaders were held at the three study sites. During these meetings, the project team explained the purpose of the study and the schedule of activities. The team listened to community concerns and worked to address them during discussions and incorporated feedback where possible. These meetings not only increased community support for the project but were essential in securing the safety of project team in the area. Engaging government stakeholders was also an important aspect of our project as we aimed to implement delivery systems that would be sustainable by the government for future work. Inception meetings were conducted with provincial stakeholders including the Health and Finance Minister of KP, Chief Secretary of KP, Director General Health of Balochistan and the Pakistani Pediatric Association. Findings from the study will be disseminated through peer-reviewed journal publications and shared with key stakeholders who have been engaged in this project including government officials, and the Pakistan Pediatric Association.

## Discussion

*Naunehal* aims to test a low-cost simplified model of working in challenging areas that has the potential to be sustainably implemented. As well, this study is one of the first to systematically engage private health practitioners in Pakistan to deliver vaccinations to the community.

Although this is not a clinical study, there has been patient-health worker interaction within the mobile health services and private sector engagement. The baseline survey and initial pre-implementation engagement with community leaders and stakeholders informed the community engagement plan, the health promotion materials and the sites and schedule of the mobile health services. At the conclusion of the study, results will be shared with the community members through community-based dissemination and stakeholder acknowledgement events. A limitation of the study is that surveys may not capture the underlying reasons as to why the receptiveness to vaccination may change.

## Supporting information

S1 File (DOCX)

## References

[1] Bank TW. World Bank Country and Lending Groups: Country Classifications 2019. Available from: https://datahelpdesk.worldbank.org/knowledgebase/articles/906519-world-bank-country-and-lending-groups.

[2] Studies NIoP. Pakistan Demographic and Health Survey 2017–2018: Key Indicators Report. Rockville, Maryland: The DHS Program ICF, 2018.

[3] ICF NIoPSNPa. Pakistan Maternal Mortality Survey: Key Indicators Report. Islamabad, Pakistan. Rockville, Maryland, USA: NIPS and ICF, 2019.

[4] Lassi ZS, Musavi NB, Maliqi B, et al. Systematic review on human resources for health interventions to improve maternal health outcomes: evidence from low-and middle-income countries. *Human resources for health* 2016;14(1):1–20. doi: 10.1186/s12960-016-0106-y 26971317 PMC4789263

[5] Kirmani S, Saleem A. Impact of COVID-19 pandemic on paediatric services at a referral centre in Pakistan: lessons from a low-income and middle-income country setting. *Archives of disease in childhood* 2020. 10.1136/archdischild-2020-319424 PMC7371476 32601083

[6] Qazi SH, Saleem A, Pirzada AN, et al. Challenges to delivering pediatric surgery services in the midst of COVID 19 crisis: experience from a tertiary care hospital of Pakistan. *Pediatric surgery international* 2020:1–7. 32691128 10.1007/s00383-020-04721-0 PMC7370874

[7] Bhutta ZA, Hafeez A, Rizvi A, et al. Reproductive, maternal, newborn, and child health in Pakistan: challenges and opportunities. *The Lancet* 2013;381(9884):2207–18. 10.1016/S0140-6736(12)61999-0 23684261

[8] Shaikh BT. Private sector in health care delivery: a reality and a challenge in Pakistan. *Journal of Ayub Medical College Abbottabad* 2015;27(2):496–98. 26411151

[9] Nishtar S, Boerma T, Amjad S, et al. Pakistan’s health system: performance and prospects after the 18th Constitutional Amendment. *The Lancet* 2013;381(9884):2193–206. doi: 10.1016/S0140-6736(13)60019-7 23684254

[10] Ataullahjan A, Ahsan H, Soofi S, et al. Eradicating Polio in Pakistan: A systematic review of programs and policies. *Expert Review of Vaccines* 2021:1–18. doi: 10.1080/14760584.2021.1915139 33896306

[11] Closser S, Jooma R, Varley E, et al. Polio eradication and health systems in Karachi: vaccine refusals in context. *Global Health Communication* 2015;1(1):32–40.

[12] Bird ST, Bogart LM. Conspiracy beliefs about HIV/AIDS and birth control among African Americans: implications for the prevention of HIV, other STIs, and unintended pregnancy. *Journal of Social Issues* 2005;61(1):109–26. doi: 10.1111/j.0022-4537.2005.00396.x 17073026

[13] Niehaus I, Jonsson G. Dr. Wouter Basson, Americans, and wild beasts: men’s conspiracy theories of HIV/AIDS in the South African Lowveld. *Medical anthropology* 2005;24(2):179–208. doi: 10.1080/01459740590933911 16019570

[14] StataCorp. 2021. *Stata Statistical Software*: *Release 17*. College Station, TX: StataCorp LLC.

[15] *The Abdul Latif Jameel Poverty Action Lab*. Abdul Latif Jameel Poverty Action Lab (J-PAL). https://www.povertyactionlab.org/

