# Supplementary material for: NAUNEHAL; Integrated immunization and MNCH interventions: A quasi-experimental study–Protocol
Source: PLoS One. 2023 Jun 29;18(6):e0287722. doi: 10.1371/journal.pone.0287722 (PMC10310005; doi:10.1371/journal.pone.0287722)
Supplement: S1 File — (DOCX) [file pone.0287722.s001.docx]

**APPENDIX A**

**Target and Control Union Councils (UCs) Data**

|  | **Quetta** | | **Peshawar** | | **Lakki Marwat** | |
| --- | --- | --- | --- | --- | --- | --- |
|  | **UC Kharotabad-1** | **UC Ward 11-A** | **UC Bhana Mari** | **UC Sheikh Junaidabad** | **UC Bakhmal Ahmedzai** | **UC Pahar Khel Thal** |
| **Population** | 67,782 | 96,359 | 23,000 | 26,106 | 44,870 | 39,432 |
| **Children <5** | 11,528 | 16,381 | 3849 | 4438 | 7179 | 6309 |
| **Public HF** | 3 | 1 | 1 | 1 | 1 | 2 |
| **Private HF** | 7 | 7 | 8 | 6 | 4 | 16 |
| **LHWs** | 0 | 0 | 8 | 8 | 0 | 0 |
| **EPI vaccinators** | 0 | 3 | 2 | 2 | 2 | 3 |

HF = Health Facilities, EPI = Expanded Programme on Immunization

**APPENDIX B**

**Vaccination Schedule of Pakistan's Expanded Programme On Immunization**

| **When** | **Age** | **Vaccines** |
| --- | --- | --- |
| At Birth | At Birth | BCG  OPV-0  Hepatitis B |
| 2^nd^ Visit | 6 weeks | OPV-1  Pneumococcal – I  Rotavirus – I  Pentavalent - I |
| 3^rd^ Visit | 10 weeks | OPV-II  Pneumococcal – II  Rotavirus – II  Pentavalent - II |
| 4^th^ Visit | 14 weeks | OPV-III  IPV- I  Pneumococcal – III  Rotavirus – III  Pentavalent - III |
| 5^th^ Visit | 9 months | MR – I  IPV – II  Typhoid |
| 6^th^ Visit | 15 months | MR - II |

OPV = Oral Polio Vaccine, IPV = Injectable Polio Vaccine, MR = Measles Rubella

## APPENDIX C – Baseline Survey Tool

***Instructions:*** *Introduce yourself and explain the reason of your visit.*

**Section A: Identification Information (HH)**

| **Sr. No.** | **Questions** | **Responses** | **Skip** |
| --- | --- | --- | --- |
| HH01 | Date of interview | \|  \|  \| / \|  \|  \| / \| 2 \| 0 \|  \|  \| \| --- \| --- \| --- \| --- \| --- \| --- \| --- \| --- \| --- \| --- \| \| D \| D \|  \| M \| M \|  \| Y \| Y \| Y \| Y \| |  |
| HH02 | Time of Interview | ___ : ___ HH : MM |  |
| HH03 | Code of Interviewer | \|  \|  \|  \|  \| \| --- \| --- \| --- \| --- \| |  |
| HH04 | Name of Interviewer |  |  |
| HH05 | Name of District | Peshawar 1  Lakki Marwat 2  Quetta 3 |  |
| HH06 | Union Council | \|  \|  \|  \| \| --- \| --- \| --- \| |  |
| HH07 | Block, Street, Colony, Village Name, city |  |  |
| HH08 | Cluster Number | \|  \|  \|  \|  \|  \|  \| \| --- \| --- \| --- \| --- \| --- \| --- \| |  |
| HH09 | Household number | \|  \| - \|  \|  \|  \| - \|  \|  \|  \| \| --- \| --- \| --- \| --- \| --- \| --- \| --- \| --- \| --- \| |  |
| HH10 | Complete Address including landmark |  |  |
| HH11 | Before progressing, take consent; did you get permission?  If permission was not granted, stop interviewing and proceed to the next household. | Yes, permission is granted 1  No, permission is denied 2 | If HH11 = 2, exit interview |
| HH12 | Name of respondent |  |  |
| HH13 | Age of respondent (In Years)  (Minimum age: 15 years) | \|  \|  \| \| --- \| --- \| | If HH13 < 15, exit interview |
| HH14 | Gender of respondent | Male 1  Female 2 |  |
| HH16 | Qualification of respondent  *Mention completed years of education; Code: 55 for uneducated and 22 for only religious studies* | Years of education |  |
| HH17 | Profession of Respondent | Professional/technical/managerial 1  Clerical 2  Business 3  Sales and services 4  Skilled manual 5  Unskilled manual 6  Domestic service 7  Agriculture 8  Student 9  Unemployed 10  Homemaker 11  Retired 12  Housewife 13  Others 96  Please Specify___________________________ |  |
| HH18 | Is respondent the head of household | Yes 1  No 2 | If yes, skip to HH21 |
| HH19 | Name of Head of Household |  |  |
| HH20 | Profession of head of household | Professional/technical/managerial 1  Clerical 2  Business 3  Sales and services 4  Skilled manual 5  Unskilled manual 6  Domestic service 7  Agriculture 8  Student 9  Unemployed 10  Homemaker 11  Retired 12  Housewife 13  Others 96  Please Specify___________________________ |  |
| HH21 | Total members |  |  |
| HH22 | Male members including male children of all ages |  |  |
| HH23 | Female members including female children of all ages |  |  |
| HH24 | Number of under-five children - Male |  |  |
| HH25 | Number of under-five children - Female |  | If HH24 + HH25 =0, exit interview |
| HH25A | Have you been living in this House / UC / area for more than six months? | Yes 1  No 2 | If HH25A is no, exit interview |
| HH26 | Result of Interview  If the interview is not complete, please inform your supervisor | Completed 1  Partially completed 2  Refused 3  No eligible respondent available 4  Locked 5  Household not found 6  No under 5 child in this household 7  Not living in this HH/ for last six months 8  Other specify 96  _________________________________ |  |

**Section CB: Child Basic Information**

**INSTRUCTION**

**Complete Section CB for all children 0-59 months in the household**

|  | Child’s | | | | | | | Mother’s | | | | | Father’s | | |
| --- | --- | --- | --- | --- | --- | --- | --- | --- | --- | --- | --- | --- | --- | --- | --- |
| CB01 | CB02 | CB03 | CB04 | CB05 | CB06 | CB15 | CB16 | CB07 | CB08 | CB09 | CB10 | CB11 | CB12 | CB13 | CB14 |
| Line no | Name | Gender  Male……1  Female..2 | DoB  DD/MM/YY  Use 98-98-9998 if not known | Age  YY - MM | Relationship with respondent  Mother 1  Father 2  Primary Caretaker  (not parents) 3  Other familymember 4 | Birth weight (kg) | Was this birth registered?  If yes with?  Not registered 1  NADRA 2  LHW 3 | Name  *Skip if CB06 = 1* | Age  *Skip if CB06 = 1* | Education  *Skip if CB06 = 1* | Profession  *Skip if CB06 = 1* | Available?  Yes…..1  No……2  Skip if CB06=1 | Name  *Skip if CB06 = 2* | Education  *Skip if CB06 = 2* | Profession  *Skip if CB06 = 2* |
|  |  |  |  |  |  |  |  |  |  |  |  |  |  |  |  |
|  |  |  |  |  |  |  |  |  |  |  |  |  |  |  |  |
|  |  |  |  |  |  |  |  |  |  |  |  |  |  |  |  |
|  |  |  |  |  |  |  |  |  |  |  |  |  |  |  |  |
|  |  |  |  |  |  |  |  |  |  |  |  |  |  |  |  |
|  |  |  |  |  |  |  |  |  |  |  |  |  |  |  |  |
|  |  |  |  |  |  |  |  |  |  |  |  |  |  |  |  |
|  |  |  |  |  |  |  |  |  |  |  |  |  |  |  |  |

**Section CS: Health & Care-seeking Behaviors**

**INSTRUCTION**

**Complete Section CS for all children in household 0-59 months (if more than one child, then use another sheet)**

| **Sr. No.** | **Questions** | **Responses** | **Skip** |
| --- | --- | --- | --- |
| CS01 | Child’s Line No |  |  |
| CS02 | Name of Child |  |  |
| CS02A | What is the relationship of child with respondent? | Father 2  Primary Caretaker (not parents) 3  Other family member 4 | Skip if CB11 is yes  Exit module if CS20A = 4 |
| CS02B | Name of the respondent |  | Skip if CS02A = 2 |
| CS03 | Has the child had diarrhea in the last two weeks? | Yes 1  No 2 | If no, skip to CS12 |
| CS04 | How much was given to drink to the child during the diarrhoea episode? | Much Less 1  Somewhat Less 2  About The Same 3  More 4  Nothing To Drink 5  Don't Know 98 |  |
| CS05 | How much was given to eat to the child during the diarrhoea episode? | Much Less 1  Somewhat Less 2  About The Same 3  More 4  Nothing to eat 5  Don't Know 98 |  |
| CS06 | Did you seek advice or treatment for diarrhoea from any source? | Yes 1  No 2 | If no skip to CSO9 |
| CS07 | Where did you seek advice or treatment for diarrhoea? | **Public Sector**  Govt. Hospital 1  RHC/MCH 2  BHU 3  Lady Health Worker 4  Other Public Sector 961  __________________________________  Private Sector  Hospital/Clinic 6  Medical Store 7  Private Doctor 8  Homeopath 9  Dispenser / Compounder 10  Hakim 11  Dai, TBA 12  CMW 13  Other Private 962  __________________________________ |  |
| CS08 | What Was Given to Treat Diarrhea  Multiple responses | ORS 1  **Pill or syrup**  Antibiotic 2  Antimotility (anti-diarrhoea) 3  Zinc 4  Unknown pill or syrup 5  **Injection**  Antibiotic injection 6  Non-antibiotic 7  Unknown injection 8  Intravenous (iv) 9  Home remedy /herbal medicine 10  Others 96  Please Specify_________________________ |  |
| CS08A | Was the child hospitalized for diarrhea? | Yes 1  No 2 | If no, skip to CS09 |
| CS08B | If yes, what was the duration of hospitalization? | __ __ days |  |
| CS09 | Why did you not seek treatment? | Fear of COVID-19 1  Confidence that child will get better himself 2  No one to accompany was available 3  Financial reasons 5  Limitations of transport 6  Others 96  Please Specify___________________________ |  |
| CS10 | Was the child given ORS? | Yes 1  No 2 | Skip if CS06 = 1 |
| CS11 | Was the child given Zinc supplements? | Yes 1  No 2 | Skip if CS06 = 1 |
| CS12 | Has the child been sick with cough in the past two weeks? | Yes 1  No 2 |  |
| CS13 | Has the child been sick with fever in the past two weeks? | Yes 1  No 2 |  |
| CS14 | Has (NAME) had fast, short, rapid breaths or difficulty breathing at any time in the  last 2 weeks? | Yes 1  No 2 | If no, then skip to CS16  If CS12,13, 14 all no, skip to IM section |
| CS15 | Was the fast or difficult breathing due to a problem in the chest or to a blocked or runny nose? | Chest only 1  Nose only 2  Both 3  Others 96  Please Specify___________________________ |  |
| CS16 | Did you seek treatment or advice from any source? | Yes 1  No 2 | If no, skip to CS19 |
| CS17 | Where did you seek advice or treatment for cough, fever or difficulty breathing? | **Public Sector**  Govt. Hospital 1  RHC/MCH 2  BHU 3  Lady Health Worker 4  Other Public Sector 961  __________________________________  Private Sector  Hospital/Clinic 6  Medical Store 7  Private Doctor 8  Homeopath 9  Dispenser / Compounder 10  Hakim 11  Dai, TBA 12  CMW 13  Other Private 962  __________________________________ |  |
| CS18 | What was given to treat cough, fever or difficulty breathing?  [Multiple Response] | **Pill or syrup**  antibiotic *2*  unknown pill or syrup 3  **Injection**  Antibiotic injection 4  non-antibiotic 5  unknown injection 6  Intravenous (iv) 7  Home remedy/herbal medicine 8  Others 96  Please Specify_________________________ |  |
| CS18A | Was the child hospitalized for Fever/Cough/difficulty breathing? | Yes 1  No 2 | If no, skip to CS19 |
| CS18B | If yes, what was the duration of hospitalization? | __ __ days |  |
| CS19 | Why did you not seek treatment? | Fear of COVID-19 1  Confidence that child will get better himself 2  No one to accompany was available 3  Financial reasons 5  Limitations of transport 6  Others 96  Please Specify___________________________ | Skip if CS16 is yes |
| CS20 | Was (NAME) ever given vitamin A supplementation? | Yes 1  No 2  Don’t know 98 | Skip if child is <6 months old |
| CS21 | When was last dosage of vitamin A supplementation given to (NAME)? | A week ago 1  One month ago 2  In last 3 months 3  In last 6 months 4  More than a year ago 5  Don’t know 98 | Skip if child is <6 months old |

**Section IM: Child Immunization**

**INSTRUCTION**

**Complete Section IM for all children in household 35 months and under (if more than one child, then use another sheet)**

| **Sr. No.** | **Questions** | **Responses** | **Skip** |
| --- | --- | --- | --- |
| IM01 | Did you ever have a vaccination card from Government or private health provider where (name)’s vaccinations are written down? | Yes 1  No 2 | If 2 skip to IM03 |
| IM02 | If yes: may I see it please?  The respondent should already have brought the card when you got permission to begin the interview. | Seen 1  Not seen 2 | If 1 skip to IM04  If 2 skip to IM08 |
| IM03 | If not, why you didn’t receive any vaccination card? | Don’t think it’s important 1  Never visited a facility 2  Could not visit a facility because of COVID-19 3  Card was not available with the health 4  The vaccinator/ facility didn’t provide the card 5  Not aware of such cards 6  Others 96  Please Specify___________________________ | All responses skip to IM08 |
| IM04 | Check and copy Date of Birth recorded on card | \|  \|  \| / \|  \|  \| / \| 2 \| 0 \|  \|  \| \| --- \| --- \| --- \| --- \| --- \| --- \| --- \| --- \| --- \| --- \| \| D \| D \|  \| M \| M \|  \| Y \| Y \| Y \| Y \| |  |
|  | **INSTRUCTION**  Please record the date of immunization for each antigen from the card   1. If the date is recorded but not readable, record 44 in day column 2. If the date is not recorded but a tick *(*🗸*)* mark is present, record 88 in day column 3. If neither date is recorded nor a tick *(*🗸*)* mark is present, prompt the mother for each such antigen and record 66 if she confirms the receipt of respective antigen by recall otherwise record 97. | |  |
| IM0501 | BCG (Vaccination for TB) | \|  \|  \| / \|  \|  \| / \| 2 \| 0 \|  \|  \| \| --- \| --- \| --- \| --- \| --- \| --- \| --- \| --- \| --- \| --- \| \| D \| D \|  \| M \| M \|  \| Y \| Y \| Y \| Y \| |  |
| IM0502 | OPV-0 (Polio drops at birth) | \|  \|  \| / \|  \|  \| / \| 2 \| 0 \|  \|  \| \| --- \| --- \| --- \| --- \| --- \| --- \| --- \| --- \| --- \| --- \| \| D \| D \|  \| M \| M \|  \| Y \| Y \| Y \| Y \| |  |
| IM0503 | OPV-1 (Polio drops at 6 weeks) | \|  \|  \| / \|  \|  \| / \| 2 \| 0 \|  \|  \| \| --- \| --- \| --- \| --- \| --- \| --- \| --- \| --- \| --- \| --- \| \| D \| D \|  \| M \| M \|  \| Y \| Y \| Y \| Y \| |  |
| IM0504 | Penta-1 (Pentavalent 1 at 6 weeks) | \|  \|  \| / \|  \|  \| / \| 2 \| 0 \|  \|  \| \| --- \| --- \| --- \| --- \| --- \| --- \| --- \| --- \| --- \| --- \| \| D \| D \|  \| M \| M \|  \| Y \| Y \| Y \| Y \| |  |
| IM0505 | PCV-1 (Pneumococcal Conjugate at 6 weeks) | \|  \|  \| / \|  \|  \| / \| 2 \| 0 \|  \|  \| \| --- \| --- \| --- \| --- \| --- \| --- \| --- \| --- \| --- \| --- \| \| D \| D \|  \| M \| M \|  \| Y \| Y \| Y \| Y \| |  |
| IM0506 | RV-1 (Rotavirus vaccine at 6 weeks) | \|  \|  \| / \|  \|  \| / \| 2 \| 0 \|  \|  \| \| --- \| --- \| --- \| --- \| --- \| --- \| --- \| --- \| --- \| --- \| \| D \| D \|  \| M \| M \|  \| Y \| Y \| Y \| Y \| |  |
| IM0507 | OPV-2 (Polio drops at 10 weeks) | \|  \|  \| / \|  \|  \| / \| 2 \| 0 \|  \|  \| \| --- \| --- \| --- \| --- \| --- \| --- \| --- \| --- \| --- \| --- \| \| D \| D \|  \| M \| M \|  \| Y \| Y \| Y \| Y \| |  |
| IM0508 | Penta-2 (Pentavalent 2 at 10 weeks) | \|  \|  \| / \|  \|  \| / \| 2 \| 0 \|  \|  \| \| --- \| --- \| --- \| --- \| --- \| --- \| --- \| --- \| --- \| --- \| \| D \| D \|  \| M \| M \|  \| Y \| Y \| Y \| Y \| |  |
| IM0509 | PCV-2 (Pneumococcal Conjugate at 10 weeks) | \|  \|  \| / \|  \|  \| / \| 2 \| 0 \|  \|  \| \| --- \| --- \| --- \| --- \| --- \| --- \| --- \| --- \| --- \| --- \| \| D \| D \|  \| M \| M \|  \| Y \| Y \| Y \| Y \| |  |
| IM0510 | RV-2 (Rotavirus vaccine at 10 weeks) | \|  \|  \| / \|  \|  \| / \| 2 \| 0 \|  \|  \| \| --- \| --- \| --- \| --- \| --- \| --- \| --- \| --- \| --- \| --- \| \| D \| D \|  \| M \| M \|  \| Y \| Y \| Y \| Y \| |  |
| IM0511 | OPV-3 (Polio drops at 14 weeks) | \|  \|  \| / \|  \|  \| / \| 2 \| 0 \|  \|  \| \| --- \| --- \| --- \| --- \| --- \| --- \| --- \| --- \| --- \| --- \| \| D \| D \|  \| M \| M \|  \| Y \| Y \| Y \| Y \| |  |
| IM0512 | Penta-3 (Pentavalent 1 at 14 weeks) | \|  \|  \| / \|  \|  \| / \| 2 \| 0 \|  \|  \| \| --- \| --- \| --- \| --- \| --- \| --- \| --- \| --- \| --- \| --- \| \| D \| D \|  \| M \| M \|  \| Y \| Y \| Y \| Y \| |  |
| IM0513 | PCV-3 (Pneumococcal Conjugate at 14 weeks) | \|  \|  \| / \|  \|  \| / \| 2 \| 0 \|  \|  \| \| --- \| --- \| --- \| --- \| --- \| --- \| --- \| --- \| --- \| --- \| \| D \| D \|  \| M \| M \|  \| Y \| Y \| Y \| Y \| |  |
| IM0514 | IPV (INJECTABLE POLIO VACCINE AT 14 WEEKS) | \|  \|  \| / \|  \|  \| / \| 2 \| 0 \|  \|  \| \| --- \| --- \| --- \| --- \| --- \| --- \| --- \| --- \| --- \| --- \| \| D \| D \|  \| M \| M \|  \| Y \| Y \| Y \| Y \| |  |
| IM0515 | Measles-1 (AT 9 months) | \|  \|  \| / \|  \|  \| / \| 2 \| 0 \|  \|  \| \| --- \| --- \| --- \| --- \| --- \| --- \| --- \| --- \| --- \| --- \| \| D \| D \|  \| M \| M \|  \| Y \| Y \| Y \| Y \| |  |
| IM0516 | Measles-2 (AT 15 months) | \|  \|  \| / \|  \|  \| / \| 2 \| 0 \|  \|  \| \| --- \| --- \| --- \| --- \| --- \| --- \| --- \| --- \| --- \| --- \| \| D \| D \|  \| M \| M \|  \| Y \| Y \| Y \| Y \| |  |
| IM07 | Check IM0501-IM0516: Are all vaccines (BCG to Measles-2) recorded? | Yes 1  No 2 | If 1 skip to IM22A  If 2 go back to IM05_1 |
|  | **INSTRUCTION**  *If IM07 = 1 go to IM22A*  *If IM07 = 2, please fill all vaccines above in IM05* | |  |
| IM08 | Has (name) ever received any vaccinations to prevent (him/her) from getting diseases, including vaccinations received in a campaign, immunization day or Child Health Day? | Yes 1  No 2  Don’t know 98 | If 2 or DK skip to IM24 |
| IM09 | Has (name) ever received a BCG vaccination against tuberculosis – that is, an injection in the arm or shoulder that usually causes a scar? | Yes 1  No 2  Don’t know 98 |  |
| IM10 | Has (*name*) ever received any vaccination drops in the mouth to protect (him/her) from polio?  Probe by indicating that the first drop is usually given at birth and later at the same time as injections to prevent other diseases. | Yes 1  No 2  Don’t know 98 | If 2 skip to IM14  If 98 skip to IM14 |
| IM11 | Were the first polio drops received in the first two weeks after birth? | Yes 1  No 2  Don’t know 98 |  |
| IM12 | How many times was the polio vaccine received at Govt/Public health facility or from outreach facility? | Number of times __ __  Don’t know 98 |  |
| IM14 | Has (name) ever received a pentavalent vaccination – that is, an injection in the thigh to prevent (him/her) from getting tetanus, whooping cough, diphtheria, hepatitis b disease, and haemophilus influenza type b?  Probe by indicating that pentavalent vaccination is sometimes given at the same time as the polio drops. | Yes 1  No 2  Don’t know 98 | If 2, skip to IM16  If 98, skip to IM16 |
| IM15 | How many times was the pentavalent vaccine received? | Number of times __ __  Don’t know 98 |  |
| IM16 | Has (name) ever received a pneumococcal conjugate vaccination – that is, an injection to prevent (him/her) from getting pneumococcal disease, including ear infections and meningitis caused by pneumococcus?  Probe by indicating that pneumococcal conjugate vaccination is sometimes given at the same time as the pentavalent vaccination | Yes 1  No 2  Don’t know 98 | If 2 skip to IM18  If 98 skip to IM18 |
| IM17 | How many times was the pneumococcal vaccine received? | Number of times __ __  Don’t know 98 |  |
| IM18 | Has (name) ever received drops for rotavirus vaccine (RV) vaccination- a vaccine given orally soon after 6 weeks birth to protect against rotavirus infections, which are the leading cause of severe diarrhoea among young children? | Yes 1  No 2  Don’t know 98 | If 2 skip to IM20  If 98 skip to IM20 |
| IM19 | How many times were the Rota vaccine drops received? | Number of times  Don’t know 98 |  |
| IM20 | Has (name) ever received an inactivated polio vaccine (IPV) – that is, a shot in the thigh at the age of 14 weeks or older - to prevent (him/her) from getting polio? | Yes 1  No 2  Don’t know 98 |  |
| IM21 | Has (name) ever received a measles injection – that is, a shot in the arm at the age of 9 months or older - to prevent (him/her) from getting measles? | Yes 1  No 2  Don’t know 98 | If 2 skip to IM23  If 98 skip to IM23 |
| IM22 | How many times was a measles injection vaccine received? | Number of times __ __  Don’t know 98 |  |
| IM22A | Has the child ever received polio drops during a national polo campaign? | Yes 1  No 2  Don’t know 98 | If no or DK, skip to IM23 |
| IM22B | How many times has the child received Polio vaccine drops during national polio campaign? | Number of times __ __  Don’t know 98 |  |
| IM22C | Did the child receive polio drops during the last national polio campaign? | Yes 1  No 2  Don’t know 98 |  |
| IM22D | The last time (name) received the polio drops, did (he/she) also get an injection to protect against polio?  Probe to ensure that both were given, drops and injection | Yes 1  No 2  Don’t know 98 |  |
| IM23 | Where is (name) usually vaccinated?  If the child is vaccinated from multiple sources, mention the usual source with higher frequency of vaccine doses. | Govt. Health facility 1  Private health facility 2  Government outreach service 3  Others 96  Please Specify___________________________ | If IM23 = 3, skip to IM25 |
| IM23A | What means of transportation do you use for visiting the facility for immunization of (name)? | By foot 1  Cycle 2  Motor Cycle/Riskshaw 3  Car / Taxi 4  Public Transport 5  Others 96  Please Specify __________________________ |  |
| IM23B | What is the time taken to reach this facility where vaccination was received? | Hours: Minutes |  |
| IM24 | Why (name) is not vaccinated?  If the child has not received all their vaccinations, ask the mother/caretaker.  Record all the reasons mentioned but do not prompt by asking specific. Encourage the mother to provide all reasons. | Place of immunization too far 1  Time of immunization not convenient 2  Mother too busy 3  Family problem including mother ill 4  Child ill, not brought 5  Child ill, brought but not vaccinated 6  Long wait 7  Rumors 8  No faith in immunization 9  Fear of side reaction 10  Time or place of immunization not known 11  Took child but no vaccine available 12  Took child but no vaccinator 13  Took child facility closed 14  Child was sick 15  Took child but not vaccination day 16  COVID-19-related (fear of going out, sickness in family) 17  Don’t know 98  Others 96  Please Specify___________________________ | Skip if IM02 = 1 or IM08 = 1 |
| IM25 | Observe BCG scar | Scar present 1  Scar absent 2  Child not available 3  Not observed 4 |  |

**Section PD: Care seeking during pregnancy and delivery**

**(To be administered to the mother of the youngest under-five child in the household)**

| Sr. No. | **Questions** | **Responses** | **Skip** |
| --- | --- | --- | --- |
| PD01 | Child’s Line Number (youngest under 5) |  |  |
| PD02 | WRA’s Name |  |  |
| PD03 | When was your last pregnancy? | 0-5.9 months back 1  6-11.9 months back 2  12-23.9 months back 3  24 months back or longer 4 |  |
| PD04 | Did you seek antenatal care during your last pregnancy?  (Excluding current pregnancy) | Yes 1  No 2  Don’t know 98 | If no, skip to PD08 |
| PD05 | From whom did you seek antenatal care? | **HEALTH PERSONNEL**  Doctor 1  Nurse 2  Midwife 3  LHV 4  **OTHER PERSON**  Dai-TBA 5  Lady Health Worker 6  Homeopath 7  Hakim 8  Dispenser/Compounder 9  Others 96  Please Specify___________________________ |  |
| PD06 | Where did you seek antenatal care? | **Home**  Respondent’s home 1  Other home 2  **Public medical sector**  Government hospital 3  Government clinic 4  Mother & child health centre 5  Other public 961  Please Specify________________________  **Private medical sector**  Private hospital 7  Private clinic 8  Private maternity home 9  other private 962  Please Specify____________________ |  |
| PD07 | How many times did you receive antenatal care during last pregnancy?  Probe to identify the number of times antenatal care was received. If a range is given, record the minimum number of times antenatal care received. | Number of times [__] [__]  Don’t Know 98 |  |
| PD08A | As part of your antenatal care during your pregnancy, were any of the following done at least once:  (Ask about each option and circle all positive responses) | Were you weighed 1  Was your BP measured 2  Did you give urine sample 3  Did you give blood sample 4  Ultrasound 5  Received counselling on nutrition 6  Received counselling on breastfeeding 7  Received counselling on family planning 8  none of the above 9  Others 96  Please Specify___________________________ |  |
| PD08 | Why did you not seek antenatal care? | Fear of COVID-19 1  Assumption that care is not needed during pregnancy 2  On the advice of a relative or friend 3  Family knew how to care for you themselves 4  Financial reasons 5  Limitations of transport 6 |  |
| PD08B | During your last pregnancy, did you receive any tetanus injections? | Yes 1  No 2  Don’t know 98 | If no skip to PD09 |
| PD08C | How many times did you receive tetanus injection? | ___ _____ No. of Times |  |
| PD09 | Have you ever taken any iron folic acid (IFA) while you were pregnant? | Yes 1  No 2 | If no skip to PD12 |
| PD10 | How often did you take IFA during pregnancy? | Daily 1  Once a week 2  Biweekly 3  Monthly 4  Rarely 5 |  |
| PD11 | How many months or days did you take IFA? | Months [__] [__]  Days [__] [__] |  |
| PD12 | Who conducted the delivery? | **HEALTH PERSONNEL**  Doctor 1  Nurse 2  Midwife 3  LHV 4  CMW 5  **OTHER PERSON**  Dai-TBA 6  Lady Health Worker 7  Others 96  Please Specify___________________________ |  |
| PD13 | During you last birth, where was the baby born? | Own home 1  Other home 2  Government Hospital 3  RHC 4  BHU 5  birth station 6  Other Public Facility 961  Please Specify_________________________  Private Hospital/Clinic 7  Other Private Facility 962  Specify________________________________ | If not 1 or 2 then skip to PD15 |
| PD14 | If at home, why was the delivery not in a facility? | Fear of COVID-19 1  All my deliveries have been at home 2  My family did not want me to go to  a facility 3  Financial reasons 4  Limitations of transport 5  Others 96  Please Specify___________________________ |  |
| PD15 | Did anyone check on your health after delivery? | Yes 1  No 2 | If no, skip to PD19 |
| PD16 | Who checked your health after delivery?  [Multiple responses] | Gynaecologist 1  Other doctor 2  Nurse 3  CMW 4  LHW 5  LHV 6  TBA 7  Others 96  Please Specify___________________________ |  |
| PD17 | How long after delivery did the first of these checks happen?  If less than one day, record hours.  If less than one week, record days.  Otherwise, record weeks. | Hours [__][__] 01  Days [__][__] 02  Weeks [__][__] 03  Don’t know/ don’t remember 98 |  |
| PD18 | How many times such check happen? | Times [__][__] |  |
| PD19 | Did anyone check on (name of child) health? | Yes 1  No 2  Don’t know 98 | If no, DK skip to PD23 |
| PD20 | Who checked the child’s health after delivery?  [Multiple responses] | Paediatrician 1  Other doctor 2  Nurse 3  CMW 4  LHW 5  LHV 6  TBA 7  Others 96  Please Specify___________________________ |  |
| PD21 | How long after delivery did the first of these checks happen?  If less than one day, record hours.  If less than one week, record days.  Otherwise, record weeks. | Hours [__][__] 01  Days [__][__] 02  Weeks [__][__] 03  Don’t know/ don’t remember 98 |  |
| PD22 | How many times such check happen? | Times [__][__] |  |
| PD23 | After the cord was cut and until it fell off, was anything applied to the cord? | Yes 1  No 2  Don’t know 98 |  |
| PD24 | What was applied to the cord?  [Multiple responses] | Chlorhexidine 1  Other antiseptic (alcohol, spirit, gentian violet, Dettol) 2  Surma 3  Mustard oil 4  Ash 5  Animal dung 6  Others 96  Please Specify___________________________  Don’t know / don’t remember 98 | skip if PD23 is no or DK |

**Section BF: Breastfeeding, Health & Care-seeking Behaviors**

Instruction: To be administered to the mother of the youngest child in the household, between 0-23 months of age

| **Sr. No.** | **Questions** | **Responses** | **Skip** |
| --- | --- | --- | --- |
| BF01 | Child’s Line Number (youngest under 2) |  |  |
| BF02 | WRA’s Name |  |  |
| BF03 | Child’s date of birth? | \|  \|  \| / \|  \|  \| / \| 2 \| 0 \|  \|  \| \| --- \| --- \| --- \| --- \| --- \| --- \| --- \| --- \| --- \| --- \| \| D \| D \|  \| M \| M \|  \| Y \| Y \| Y \| Y \| |  |
| BF03A | Child’s age | Months _____  Years _______ |  |
| BF04 | Has (NAME) ever been breastfed? | Yes 1  No 2  Don’t know 98 | If no, or don’t know skip to BF11 |
| BF05 | How long after birth did you first put (NAME) to the breast? | 1. Immediately___ OR 2. Hours _____ OR 3. Days _____ OR |  |
| BF06 | Did you feed first milk (colostrum) to the child? | Yes 1  No 2  Don’t know 98 | If yes, skip to BF08 |
| BF07 | Why did you not feed colostrum to (NAME)? | Bad milk 1  Harmful for child 2  Thickness 3  Others 96  Please Specify___________________________ |  |
| BF08 | Was (NAME) given anything to drink before breast milk? | Yes 1  No 2  Don’t know 98 | If no or don’t know then skip to BF10 |
| BF09 | What was (NAME) given to drink? | Milk (other than breast milk) 1  Plain water 2  Sugar or glucose water 3  Gripe water 4  Sugar-salt-water solution 5  Fruit juice 6  Infant formula 7  Tea / Infusions / Traditional herbal preparations 8  Honey 9  Prescribed medicine 10  Others 96  Please Specify___________________________  Not given anything to drink 99 |  |
| BF10 | Was (NAME) breastfed yesterday during the day or at night? | Yes 1  No 2  Don’t know 98 | If yes, skip to BF12 |
| BF11 | Sometimes babies are fed breast milk in different ways, for example by spoon, cup, or bottle. This can happen when the mother cannot always be with her baby. Sometimes babies are breastfed by another woman, or given breast milk from another woman by spoon, cup or bottle or some other way. This can happen if a mother cannot breastfeed her own baby.  Did (NAME) consume breast milk in any of these ways yesterday during the day or at night? | Yes 1  No 2  Don’t know 98 |  |
| BF12 | Now I would like to ask you about some medicines and vitamins that are sometimes given to infants.  Was (NAME) given any vitamin drops or other medicines as drops yesterday during the day or at night? | Yes 1  No 2  Don’t know 98 |  |
| BF13 | Was (NAME) given ORS/NIMKOL yesterday during the day or at night? | Yes 1  No 2  Don’t know 98 |  |
| BF14 | Next I would like to ask you about some liquids that (NAME) may have had yesterday during the day or at night.  Did (NAME) have any (ITEM FORM LIST)?  READ THE LIST OF LIQUIDS STARTING WITH ‘PLAN WATER’ | Plain water Y/N/DK  Infant formula Y/N/DK  Infant formula Times __  Milk such as tinned, powdered, or fresh animal milk Y/N/DK  Milk such as tinned, powdered, or fresh animal milk . Times __  Juice or juice drinks Y/N/DK  Clear broth Y/N/DK  Yogurt Y/N/DK  Yogurt Times __  Thin porridge Y/N/DK  Any other liquids such as [list other water-based liquids available in the local setting] Y/N/DK  Any other liquids Y/N/DK |  |
| BF15 | Please describe everything that (NAME) ate yesterday during the day or night, whether at home or outside the home. | Porridge, bread, rice, noodles, or other foods made from grains Y/N/DK  Pumpkin, carrots, squash, or sweet potatoes that are yellow or orange inside Y/N/DK  White potatoes, white yams manioc, cassava, or any other food made from roots Y/N/DK  Any dark green leafy vegetables Y/N/DK  Ripe mangoes, ripe papayas Y/N/DK  Any other fruits or vegetables Y/N/DK  Liver, kidney, heart, or other organ meats Y/N/DK  Any meat, such as beef, lamb, goat chicken, or duck Y/N/DK  Eggs Y/N/DK  Fresh or dried fish, shellfish, or seafood Y/N/DK  Any food made from beans, peas lentils, nuts, or seeds Y/N/DK  Cheese, yogurt, or other milk product Y/N/DK  Any oil, fats, or butter, or foods made with any of these Y/N/DK  Any sugary foods such as chocolates, sweets candies, pastries cakes, or biscuits Y/N/DK  Condiments for flavor, such as chilies, spices, herbs, or fish powder Y/N/DK  Foods made with red palm oil, red palm nut, or red palm nut pulp sauce Y/N/DK |  |
|  | Check responses in BF15, if all ‘no’ for BF15, go BF16. If at least one ‘yes’ or all ‘DK’ go to BF17 | |  |
| BF16 | Did (NAME) eat any solid, semi- solid, or soft foods yesterday during the day or at night?  IF ‘YES’ PROBE: what kind of solid, semi-solid, or soft foods did (NAME) eat?  If BF16 is “yes” & all responses to BF15 are “no” please go back to BF15 and record foods items consumed. Then continue with BF17. | Yes 1  No 2  Don’t know 98 | If no or DK, skip to BF18 |
| BF17 | How many times did (NAME) eat solid, semi-solid, or soft foods other than liquids yesterday during the day or at night? | Number of times [__][__]  Don’t know 98 |  |
| BF18 | Did (NAME) drink anything from a bottle with a nipple yesterday during the day or night? | Yes 1  No 2  Don’t know 98 | If no, skip to BF20 |
| BF19 | What did (name) drink from the bottle with a nipple? | Top feed (formula milk) 1  Animal milk 2  Expressed milk 3  Others 96  Please Specify ___________________________ |  |
| BF20 | Did (name) drink or eat vitamin or mineral supplements or any medicines yesterday, during the day or night? | Yes 1  No 2  Don’t know 98 |  |

**Section CV: COVID (To be administered to the mother of the youngest under-five child in the household)**

| **Sr. No.** | **Questions** | **Responses** | **Skip** |
| --- | --- | --- | --- |
| CV01A | Name of the respondent |  |  |
| CV01 | Have you heard of COVID-19? | Yes 1  No 2 | If no, skip to section SE |
| CV02 | How concerned are you about COVID-19? | Not Worried 1  A Little Worried 2  Somewhat Worried 3  Very Worried 4  Extremely Worried 5 |  |
| CV03 | Do you think COVID-19 exists? | Yes, it exists 1  No, it doesn’t 2  Don’t know 98 |  |
| CV04 | How deadly do you think COVID-19 is? | Not At All Deadly 1  Not Very Deadly 2  Not Deadly 3  Somewhat Deadly 4  Extremely Deadly 5  Don’t know 98 |  |
| CV05 | Do you know which group of people are most at risk of COVID?  [Multiple Responses] | Everyone 1  Elderly 2  Adults 3  Children 4  Pregnant/Lactating Women 5  Health Workers 6  Person With Pre-Existing Conditions 7  Others 96  Please Specify___________________________ |  |
| CV06 | Who do you trust for information around COVID?  (Choose all that apply. Do not read the answers, let the respondent answer spontaneously)  [Multiple Responses] | Community Leader 1  Religious Leader 2  Relatives 3  Friends 4  Health Worker At Health Facility 5  Health Worker Via Door To Door 6 Campaign 7  Newspaper 8  Radio 9  Television 10  Social Media 11  Others 96  Please Specify___________________________ |  |
| CV07 | Will all people with COVID-19 show symptoms? | Yes 1  No 2  Don’t know 98 |  |
| CV08 | What symptoms are most commonly reported with COVID-19 (check all that apply)  [Multiple Responses] | Fever 1  Cough 2  Flu 3  Sore throat 4  Chest congestion/Difficulty in breathing 5  Losing sense of smell & taste 6  Diarrhea 7  Don’t know 98  Others 96  Please Specify___________________________ |  |
| CV09 | What do you think are the different modes of transmission of COVID-19?  (Choose all that apply. Do not read the answers, let the respondent answer spontaneously)  [Multiple Responses] | Touching surfaces contaminated with the virus 1  Shaking hands 2  Directly coming in contact with an infected person’s body fluids such as blood, vomit, or sweat 3  Via droplets entering through mouth or nose when an infected person sneezes or coughs 4  Unhygienic food preparation 5  Radio waves such as mobile networks 6  Mosquito bite 7  Don’t know 98  Others 96  Please Specify___________________________ |  |
| CV10 | What according to you are effective methods of preventing infection from COVID-19?  (Choose all that apply. Do not read the answers, let the respondent answer spontaneously)  [Multiple Responses] | Frequently washing hands using soap and water for 20 seconds 1  Cleaning hands with hand sanitizer 2  Covering mouth and nose using a tissue or flexed elbow while coughing or sneezing 3  Maintain social distancing 4  Avoiding contact with sick people showing signs of COVID-19 5  Wearing a mask in public places 6  Keep oneself isolated 7  Praying to God 8  Don’t know 98  Others 96  Please Specify___________________________ |  |
| CV11 | Has COVID-19, the subsequent lockdown & restrictions and fear affected the nutrition and feeding of your child? | Yes 1  No 2 | If no, skip to CV13 |
| CV12 | How has it affected breastfeeding and/or feeding of the child?  (Choose all that apply. Do not read the answers, let the respondent answer spontaneously) | I have reduced the frequency and amount I feed my child 1  I have increased the frequency and amount I feed my child 2  I cannot afford same food for child 3  I cannot travel to markets to purchase food 4  The foods I feed my child are no longer available in the market 5  Others 96  Please Specify___________________________ |  |
| CV13 | Has COVID-19, the subsequent lockdown & restrictions and fear disrupted your child’s immunization? | Yes 1  No 2 |  |
| CV14 | Has your child (under-5) received oral polio vaccine in the past 10 months? | Yes 1  No 2 |  |
| CV15 | Has your child received all vaccinations in the past 10 months that were due as per EPI schedule? | Yes 1  No 2 |  |
| CV16 | If not, why was the child not taken for vaccination? | Fear of COVID-19 1  Financial reasons 2  Limitations of transport 3  Vaccinations can be delayed 4  Forgot that there was a vaccine due 5  Interrupted services 6  Others 96  Please Specify___________________________ |  |
| CV17 | Has the number of times you visit health facilities reduced during COVID-19? | Yes 1  No 2 | If no, skip to CV19 |
| CV18 | Why has it decreased? | Fear of COVID-19 1  Financial reasons 2  Limitations of transport 3  Vaccinations can be delayed 4  Forgot that there was a vaccine due 5  Interrupted services 6  Others 96  Please Specify___________________________ |  |
| CV19 | For what conditions have you decided to not go to the doctor? | My child had diarrhea 1  My child had fever or cough 2  My/my wife’s pregnancy and/or childbirth 3  My child’s health issue, prefer not specify 4  My health, prefer not to specify 5  Other, prefer not to say 6  Others 96  Please Specify___________________________ |  |

**Section SE: Household Characteristics**

| **Sr. No.** | **Questions** | **Responses** | **Skip** |
| --- | --- | --- | --- |
| SE01 | What is the mother tongue of the head of the household? | Urdu 1  Punjabi 2  Sindhi 3  Pushtu 4  Baluchi 5  Siraiki 6  Hindko 7  Persian 8  Brohi 9  Others 96  Please Specify___________________________ |  |
| SE02 | Main material of the dwelling floor | **Natural Floor**  Mud/Clay/Earth/Sand 1  Dung 2  **Rudimentary/Floor**  Wood planks 3  Palm/Bamboo 4  **Finished Floor**  Parquet or polished wood 5  Vinyl or asphalt strips 6  Ceramic tiles 7  Cement 8  Carpet 9  Bricks 10  Lime 11  Others 96  Please Specify___________________________ |  |
| SE03 | What is the primary construction material used to build the house (by observation)? | **NATURAL WALLS**  No Walls 1  Cane/Palm/Trunks 2 Dirt 3  Mud/Stones 4  Bamboo/Sticks/Mud 5  **RUDIMENTARY WALLS**  Unbaked Bricks/Mud 6  Bamboo With Mud 7  Stone With Mud 8  Uncovered Adobe 9  Plywood 10  Reused Wood 11  **FINISHED WALLS**  Cement 12  Stone With Lime/Cement 13  Bricks 14  Cement Blocks 15  Covered Adobe 16  Wood Planks/Shingles 17  Others 96  Please Specify___________________________ |  |
| SE04 | Observe the primary construction material  Used to build the roof? | **NATURAL ROOFING**  No Roof 1  Thatch/Palm Leaf 2  Sod/Grass 3  **RUDIMENTARY ROOFING**  Rustic Mat 4  Palm/Bamboo 5  Wood Planks 6  Cardboard 7  **FINISHED ROOFING**  Asbestos 8  Reinforced Brick Cement/R 9  Metal 10  Wood 11  Calamine/Cement Fiber 12  Ceramic Tiles 13  Cement/RCC 14  Roofing Shingles 15  Others 96  Please Specify ______________________ |  |
| SE05 | What is the ownership status of this house? | Owned 1  Rented 2  Living without paying rent 3  Others 96  Please Specify___________________________ | If the اnswer Is 1,3 OR 96, skip to SE07 |
| SE06 | If this house is rental, what is the monthly rent of this house? | Rupees: ______________________________ |  |
| SE07 | How many rooms in this HH are used for sleeping? | number: |  |
| SE08 | What is the main source of drinking water  In this HH? | **PIPED WATER**  Piped Into Dwelling 1  Piped To Yard/Plot 2  Piped To Neighbor 3  Public Tap/Standpipe 4  **DUG WELL / BORING**  Tube Well / Borehole /Hand pump 5  Protected Well 6  Unprotected Well 7  **WATER FROM SPRING**  Protected Spring 8  Unprotected Spring 9  Rainwater 10  Tanker Truck 11  Cart With Small Tank 12  Surface Water (River/Dam/  Lake/Pond/Stream/Canal/  Irrigation Channel) 13  Bottled Water 14  Others 96  Please Specify___________________________ |  |
| SE09 | Do you do anything to the water to make it safer to drink? | Yes 1  No 2  Don’t know 98 | If no, skip to SE11 |
| SE10 | What do you usually do to make the water safer to drink?  Anything else?  RECORD ALL MENTIONED. | Boil 1  Add Bleach/Chlorine 2  Strain Through A Cloth 3  Use Water Filter (Ceramic/  Sand/Composite/Etc) 4  Solar Disinfection 5  Let It Stand And Settle 6  Other 96  Pleas Specify___________________________  Don't Know 98 |  |
| SE11 | What kind of toilet facility do members of your HH usually use? | **FLUSH OR POUR FLUSH TOILET**  Flush To Piped Sewer System 1  Flush To Septic Tank 2  Flush To Pit Latrine 3  Flush To Somewhere Else 4  Flush, Don't Know Where 5  **PIT LATRINE**  Ventilated Improved Pit Latrine 6  Pit Latrine With Slab 7  Pit Latrine Without Slab/Open Pit 8  Composting Toilet 9  Bucket Toilet 10  Hanging Toilet/Hanging Latrine 11  No Facility/Bush/Field 12  Others 96  Please Specify___________________________ |  |
| SE12 | What is the drainage system of this HH?  (Through observation) | Underground sewerage lines 1  Open sewerage line 2  Unattached sewerage lines 3  Others 96  Please Specify___________________________ |  |
| SE13 | We would like to learn about the places that households use to wash their hands. Can you please show me where members of your household most often wash their hands? | Observed, Fixed Place 1  Observed, Mobile 2  **NOT OBSERVED**  Not in Dwelling/Yard/Plot 3  Not Observed, No Permission to See 4  Not Observed, Other Reason 5 | If not observed,  then skip to SE16 |
| SE14 | Observe presence of water at the place of washing  *RECORD OBSERVATION* | Water Is Available 1  Water Is Not Available 2 |  |
| SE15 | Observe presence of soap, detergent, or other cleansing agent at the place for handwashing.  *RECORD OBSERVATION* | Soap or Detergent (Bar, Liquid, Powder, Paste) 1  Ash, Mud, Sand 2  None 3 |  |
| SE16 | Do you have any soap or detergent in your household for hand washing? | Yes 1  No 2 | If no, skip to SE18 |
| SE17 | If yes, can you please show it to me?  [Multiple responses] | Bar soap 1  Detergent (powder/liquid/paste) 2  Liquid soap 3  Ash/mud/sand 4  None 5 |  |
| SE18 | In what situations do you wash your hands with soap?  *This is an open-ended question. Do not read the answer choices.* | 1. Before preparing food Y / N 2. Before eating Y / N 3. Before feeding a child Y / N 4. After handling feces/diapers Y / N 5. After defecating or using the latrine Y / N   96. Others Y / N  Please Specify ______________________ |  |
| SE19 | What fuel does your HH use for cooking? | Electricity 1  LPG 2  Natural Gas 3  Biogas 4  Kerosene 5  Coal, Lignite 6  Charcoal 7  Wood 8  Straw/Shrubs/Grass 9  Agricultural Crop 10  Animal Dung 11  No Food Cooked In Household 12  Others 96  Please Specify ______________________ |  |
| SE20 | What transport does most of the family members  of this HH usually use? (select all that apply) | Private car 1  Company car 2  Motorcycle Public transport 3  By walk 4  Cycle 5  Others 96  Please Specify ______________________ |  |
| SE21 | What methods do you use to protect from flies and mosquitos?  [Multiple responses] | Spray 1  Mat 2  Mosquito net 3  Globe 4  Smoke Or frankincense 5  Others 96  Please Specify ______________________ |  |
| SE22 | Does your household have | Electricity Y/N  Radio Y/N  Television Y/N  Non-Mobile Telephone Y/N  Refrigerator Y/N  Almirah/Cabinet Y/N  Chair Y/N  Room Cooler Y/N  Air conditioner Y/N  Washing Machine Y/N  Water Pump Y/N  Bed Y/N  Clock Y/N  Sofa Y/N  Camera Y/N  Sewing Machine Y/N  Computer /laptop Y/N  Internet Connection Y/N |  |
| SE23 | Does anyone in your household own a mobile phone? | Yes 1  No 2 | If no, then  Skip to SE31 |
| SE24 | How many mobile phones are there in your household? |  |  |
| SE25 | How many of these are smartphones? |  | If none, then skip  To SE31 |
| SE26 | How many of these smartphones are Android-based? |  | If none, then skip  SE31 |
| SE27 | Which mobile service provider is the Android-based smartphone connected to?  *[Multiple responses]* | Ufone 1  Jazz 2  Zong 3  Telenor 4 |  |
| SE28 | What connectivity speed does the Android-based smartphone have?  *[Multiple responses]* | 2G 1  3G 2  4G 3 |  |
| SE29 | Does the caregiver of a young child in the household have access to the Android smartphone or not? | Yes 1  No 2 |  |
| SE30 | Who owns their own Android smart phone?  *[Multiple responses]* | Myself My mother-in-law/mother 1  My husband/wife My father-in-law/father 2  Children 3  Others 96  Please Specify___________________________ |  |
| SE31 | Where do your family members usually go to seek care? | Govt. Health care facility 1  Private health care facility 2  Others 96  Please Specify___________________________ |  |
| SE32 | From whom do your family members usually seek care? | **HEALTH PERSONNEL**  Doctor 1  Nurse 2  Midwife 3  LHV 4  **OTHER PERSON**  Dai-TBA 5  Lady Health Worker 6  Homeopath 7  Hakim 8  Dispenser/Compounder 9 |  |
| SE33 | How much time does it take to reach any govt or private health care facility near your house by walking? | Hours Minutes |  |
| SE34 | For how long you had been living in this house? | Months: Years: |  |
| SE35 | Do you have any plans to shift somewhere else next year? | Yes 1  No 2  Don’t know 98 |  |
| SE36 | Has your household or anyone in your household received financial assistance in last 12 months? | Yes 1  No 2  Don’t know 98 | If no skip to SE40 |
| SE37 | If yes, then please identify which program they are registered with?  *[Multiple Responses]* | Unconditional Cash Transfer 1  Waseela-e-Haq 2  Waseela-e-Taleem 3  Waseela-e-Rozgar 4  Waseela-e-Sehat Bait ul Mal 5  EOBI 6  BISP 7  Workers Welfare Funds 8  National Income Support Program 9  Ehsas program 10  Others 96  Please Specify___________________________ |  |
| SE38 | When was the last payment received from this program? | In the last one month 1  In the last 03 months 2  In the last 06 months 3  More than one year ago 4  More than two years ago 5  Don’t know 98 |  |
| SE39 | Is a financial assistance program receipt available?  Observed by surveyor.  RECORD OBSERVATION | Available and observed 1  Not observed 2 |  |
| SE40 | Have you or anyone in the household received a food supplement from an organization in the past 12 months? | Yes 1  No 2  Don’t know 98 |  |

## APPENDIX D – Endline Survey Tool

***Instructions:*** *Introduce yourself and explain the reason of your visit.*

**Section A: Identification Information (HH)**

| **Sr. No.** | **Questions** | **Responses** | **Skip** |
| --- | --- | --- | --- |
| HH01 | Date of interview | \|  \|  \| / \|  \|  \| / \| 2 \| 0 \|  \|  \| \| --- \| --- \| --- \| --- \| --- \| --- \| --- \| --- \| --- \| --- \| \| D \| D \|  \| M \| M \|  \| Y \| Y \| Y \| Y \| |  |
| HH02 | Time of Interview | ___ : ___ HH : MM |  |
| HH03 | Code of Interviewer |  |  |
| HH04 | Name of Interviewer |  |  |
| HH05 | Name of District | Peshawar 1  Lakki Marwat 2  Quetta 3 |  |
| HH06 | Union Council |  |  |
| HH07 | Block, Street, Colony, Village Name |  |  |
| HH08 | Cluster Number |  |  |
| HH09 | Household number |  |  |
| HH10 | Complete Address including landmark |  |  |
| HH11 | Before progressing, take consent; did you get permission?  If permission was not granted, stop interviewing and proceed to the next household. | Yes, permission is granted 1  No, permission is denied 2 | If HH11 = 2, exit interview |
| HH12 | Name of respondent |  |  |
| HH13 | Age of respondent (In Years)  (Minimum age: 18 years) |  | If HH13 < 18, exit interview |
| HH14 | Gender of respondent | Male 1  Female 2 |  |
| HH15 | Literacy level of respondent:  Now I would like you to read this sentence to me.  *SHOW CARD TO RESPONDENT*  *If respondent cannot read whole sentence,*  *PROBE: Can you read any part of the sentence to me?* | Cannot Read at All 1  Able to Read Only Part of The Sentence………..2  Able to Read Whole Sentence 3  No Card With required language 4  Blind/Visually Impaired 5 |  |
| HH16 | Qualification of respondent  *Mention completed years of education; Code: 55 for uneducated, 17 for Professional and 22 for only religious studies* | Years of education |  |
| HH17 | Profession of Respondent | Professional/technical/managerial 1  Clerical 2  Business 3  Sales and services 4  Skilled manual 5  Unskilled manual 6  Domestic service 7  Agriculture 8  Student 9  Unemployed 10  Homemaker 11  Retired 12  Housewife 13  Others 96  Please Specify___________________________ |  |
| HH18 | Is respondent the head of household | Yes 1  No 2 | If yes, skip to HH21 |
| HH19 | Name of Head of Household |  |  |
| HH20 | Profession of head of household | Professional/technical/managerial 1  Clerical 2  Business 3  Sales and services 4  Skilled manual 5  Unskilled manual 6  Domestic service 7  Agriculture 8  Student 9  Unemployed 10  Homemaker 11  Retired 12  Housewife 13  Others 96  Please Specify___________________________ |  |
| HH21 | Total members |  |  |
| HH22 | Male members including male children of all ages |  |  |
| HH23 | Female members including female children of all ages |  |  |
| HH24 | Number of under-five children - Male |  |  |
| HH25 | Number of under-five children - Female |  |  |
| HH25A | Have you been living in this House / UC / area for more than six months? | Yes 1  No 2 | If HH25A is no,  exit interview |
| HH26 | Result of Interview | Completed 1  Partially completed 2  Refused 3  No eligible respondent available 4  Locked 5  Household not found 6  No under-5 child in this household 7  Not living in this HH/ for last six months 8  Other specify 96  _________________________________ |  |

**Section CB: Child Basic Information**

**INSTRUCTION**

**Complete Section CB for all children in household 59 months and under (if more than one child, then use another sheet)**

|  | Child’s | | | | | Mother’s | | | | | Father’s | | | Child’s | |
| --- | --- | --- | --- | --- | --- | --- | --- | --- | --- | --- | --- | --- | --- | --- | --- |
| CB01 | CB02 | CB03 | CB04 | CB05 | CB06 | CB07 | CB08 | CB09 | CB10 | CB11 | CB12 | CB13 | CB14 | CB15 | CB16 |
| Line no | Name | Gender  Male……1  Female..2 | DoB  DD/MM/YY  Use 98 if not known | Age  YY - MM | Relationship with respondent  Mother 1  Father 2  Primary Caretaker  (not parents) 3  Other familymember 4 | Name  *Skip if CB06 = 1* | Age  *Skip if CB06 = 1* | Education  *Skip if CB06 = 1* | Profession  *Skip if CB06 = 1* | Available?  Yes…..1  No……2 | Name  *Skip if CB06 = 2* | Education  *Skip if CB06 = 2* | Profession  *Skip if CB06 = 2* | Birth weight (kg) | Was this birth registered?  If yes with?  Not registered 1  NADRA 2  LHW 3 |
|  |  |  |  |  |  |  |  |  |  |  |  |  |  |  |  |
|  |  |  |  |  |  |  |  |  |  |  |  |  |  |  |  |
|  |  |  |  |  |  |  |  |  |  |  |  |  |  |  |  |
|  |  |  |  |  |  |  |  |  |  |  |  |  |  |  |  |
|  |  |  |  |  |  |  |  |  |  |  |  |  |  |  |  |
|  |  |  |  |  |  |  |  |  |  |  |  |  |  |  |  |
|  |  |  |  |  |  |  |  |  |  |  |  |  |  |  |  |
|  |  |  |  |  |  |  |  |  |  |  |  |  |  |  |  |

**Section CS: Health & Care-seeking Behaviors**

**INSTRUCTION**

**Complete Section CS for all children in household 0-59 months (if more than one child, then use another sheet)**

| **Sr. No.** | **Questions** | **Responses** | **Skip** |
| --- | --- | --- | --- |
| CS01 | Child’s Line No |  |  |
| CS02 | Name of Child |  |  |
| CS03 | Has the child had diarrhea in the last two weeks? | Yes 1  No 2 | If no, skip to CS11 |
| CS04 | How much was given to drink to the child during the diarrhoea episode? | Much Less 1  Somewhat Less 2  About The Same 3  More 4  Nothing To Drink 5  Don't Know 98 |  |
| CS05 | How much was given to eat to the child during the diarrhoea episode? | Much Less 1  Somewhat Less 2  About The Same 3  More 4  Never Gave Food 5  Don't Know 98 |  |
| CS06 | Did you seek advice or treatment for the diarrhoea from any source? | Yes 1  No 2 | If no skip to CS09 |
| CS07 | Where did you seek advice or treatment for diarrhoea? | **Public Sector**  Govt. Hospital 1  RHC/MCH 2  BHU 3  Lady Health Worker 4  Other Public Sector 961  __________________________________  Private Sector  Hospital/Clinic 6  Medical Store 7  Pvt. Doctor 8  Homeopath 9  Dispenser / Compounder 10  Hakim 11  Dai, TBA 12  CMW 13  Naunehalhealth camp…………………………………14  Other Private 962  __________________________________ |  |
| CS08 | What Was Given to Treat Diarrhea  Multiple responses | ORS 1  **Pill or syrup**  Antibiotic 2  Antimotility (anti-diarrhoea) 3  Zinc 4  Unknown pill or syrup 5  **Injection**  Antibiotic 6  Non-antibiotic 7  Unknown injection 8  Intravenous (iv) 9  Home remedy /herbal medicine 10  Others 96  Please Specify_________________________ |  |
| CS09 | Why did you not seek treatment | Fear of COVID-19 1  Confidence that child will get better himself 2  Someone you knew advised treatment 3  Family knew management themselves 4  Financial reasons 5  Limitations of transport 6  Others 96  Please Specify___________________________ |  |
| CS10 | Was the child given ORS? | Yes 1  No 2 | Skip if CS06 = 1 |
| CS11 | Was the child given Zinc supplements? | Yes 1  No 2 | Skip if CS06 = 1 |
| CS12 | Has the child been sick with cough in the past two weeks? | Yes 1  No 2 |  |
| CS13 | Has the child been sick with fever in the past two weeks? | Yes 1  No 2 |  |
| CS14 | Has (NAME) had fast, short, rapid breaths or difficulty breathing at any time in the  last 2 weeks? | Yes 1  No 2 | If no, then skip to CS16  If CS12,13, 14 all no, skip to IM section |
| CS15 | Was the fast or difficult breathing due to a problem in the chest or to a blocked or runny nose? | Chest only 1  Nose only 2  Both 3  Others 96  Please Specify___________________________ |  |
| CS16 | Did you seek treatment from any source? | Yes 1  No 2 | If no, skip to CS19 |
| CS17 | Where did you seek advice or treatment for cough, fever or difficulty breathing? | **Public Sector**  Govt. Hospital 1  RHC/MCH 2  BHU 3  Lady Health Worker 4  Other Public Sector 961  __________________________________  Private Sector  Hospital/Clinic 6  Medical Store 7  Pvt. Doctor 8  Homeopath 9  Dispenser / Compounder 10  Hakim 11  Dai, TBA 12  CMW 13  Naunehalhealth camp…………………………………14  Other Private 962  __________________________________ |  |
| CS18 | What Was Given to Treat cough, fever or difficulty breathing? | **Pill or syrup**  antibiotic *2*  unknown pill or syrup 3  **Injection**  antibiotic 4  non-antibiotic 5  unknown injection 6  Intravenous (iv) 7  Home remedy/herbal medicine 8  Others 96  Please Specify_________________________ |  |
| CS17A | Was the child hospitalized for Fever/Cough/difficulty breathing? | Yes ................................................................... 1  No .................................................................... 2 | If no, skip to CS19 |
| CS17B | If yes, what was the duration of hospitalization? | __ __ days |  |
| CS19 | Why did you not seek treatment? | Fear of COVID-19 1  Confidence that child will get better himself2  Someone you knew advised treatment 3  Family knew management themselves 4  Financial reasons 5  Limitations of transport 6  Others 96  Please Specify___________________________ | Skip if CS16 is yes |
| CS20 | Was (NAME) ever given vitamin A supplementation? | Yes -------------------------------------------------------- 1  No -------------------------------------------------------- 2  Don’t know ................................................... 98 | Skip if child is <6 months old |
| CS21 | When was last dosage of vitamin A supplementation given to (NAME)? | A week ago ---------------------------------------------- 1  One month ago ---------------------------------------- 2  In last 3 months ---------------------------------------- 3  In last 6 months ---------------------------------------- 4  More than a year ago -------------------------------- 5  Don’t know -------------------------------------------- 98 | Skip if child is <6 months old |

**Section IM: Child Immunization**

**INSTRUCTION**

**Complete Section IM for all children in household 35 months and under (if more than one child, then use another sheet)**

| **Sr. No.** | **Questions** | **Responses** | **Skip** |
| --- | --- | --- | --- |
| IM01 | Did you ever have a vaccination card from a Government or private health provider where (name)’s vaccinations are written down? | Yes 1  No 2 | If 2 skip to IM03 |
| IM02 | If yes: may I see it please?  The respondent should already have brought the card when you got permission to begin the interview. | Seen 1  Not seen 2 | If 1 skip to IM04  If 2 skip to IM02A |
| IM02A | Reason if the card was not seen | Card not found at this time ................1  Card Misplaced ...................................2 Card is at vaccination center/with vaccinator............................................3 Other Specify.......................................9 | All responses skip to IM08 |
| IM03 | If not, why you didn’t receive any vaccination card? | Don’t think it’s important 1  Never visited a facility 2  Could not visit a facility because of COVID-19 3  Card was not available with the health 4  The vaccinator/ facility didn’t provide the card 5  Not aware of such cards 6  Others 96  Please Specify___________________________ | All responses skip to IM08 |
| IM04 | Check and copy Date of Birth recorded on card | \|  \|  \| / \|  \|  \| / \| 2 \| 0 \|  \|  \| \| --- \| --- \| --- \| --- \| --- \| --- \| --- \| --- \| --- \| --- \| \| D \| D \|  \| M \| M \|  \| Y \| Y \| Y \| Y \| |  |
|  | **INSTRUCTION**  Please record the date of immunization for each antigen from the card   1. If the date is recorded but not readable, record 44 in day column 2. If the date is not recorded but a tick *(*🗸*)* mark is present, record 88 in day column 3. If neither date is recorded nor a tick *(*🗸*)* mark is present, prompt the mother for each such antigen and record 66 if she confirms the receipt of respective antigen by recall otherwise record 97. | |  |
| At Birth | | | |
| IM0501 | BCG (Vaccination for TB) | \|  \|  \| / \|  \|  \| / \| 2 \| 0 \|  \|  \| \| --- \| --- \| --- \| --- \| --- \| --- \| --- \| --- \| --- \| --- \| \| D \| D \|  \| M \| M \|  \| Y \| Y \| Y \| Y \| |  |
| IM0502 | OPV-0 (Polio drops at birth) | \|  \|  \| / \|  \|  \| / \| 2 \| 0 \|  \|  \| \| --- \| --- \| --- \| --- \| --- \| --- \| --- \| --- \| --- \| --- \| \| D \| D \|  \| M \| M \|  \| Y \| Y \| Y \| Y \| |  |
| IM0502A | Hepatitis B (Vaccination) | \|  \|  \| / \|  \|  \| / \| 2 \| 0 \|  \|  \| \| --- \| --- \| --- \| --- \| --- \| --- \| --- \| --- \| --- \| --- \| \| D \| D \|  \| M \| M \|  \| Y \| Y \| Y \| Y \| |  |
| at 6 weeks of age | | | |
| IM0503 | OPV-1 (Polio drops at 6 weeks) | \|  \|  \| / \|  \|  \| / \| 2 \| 0 \|  \|  \| \| --- \| --- \| --- \| --- \| --- \| --- \| --- \| --- \| --- \| --- \| \| D \| D \|  \| M \| M \|  \| Y \| Y \| Y \| Y \| |  |
| IM0504 | Penta-1 (Pentavalent 1 at 6 weeks) | \|  \|  \| / \|  \|  \| / \| 2 \| 0 \|  \|  \| \| --- \| --- \| --- \| --- \| --- \| --- \| --- \| --- \| --- \| --- \| \| D \| D \|  \| M \| M \|  \| Y \| Y \| Y \| Y \| |  |
| IM0505 | PCV-1 (Pneumococcal Conjugate at 6 weeks) | \|  \|  \| / \|  \|  \| / \| 2 \| 0 \|  \|  \| \| --- \| --- \| --- \| --- \| --- \| --- \| --- \| --- \| --- \| --- \| \| D \| D \|  \| M \| M \|  \| Y \| Y \| Y \| Y \| |  |
| IM0506 | RV-1 (Rotavirus vaccine at 6 weeks) | \|  \|  \| / \|  \|  \| / \| 2 \| 0 \|  \|  \| \| --- \| --- \| --- \| --- \| --- \| --- \| --- \| --- \| --- \| --- \| \| D \| D \|  \| M \| M \|  \| Y \| Y \| Y \| Y \| |  |
| IM0507 | OPV-2 (Polio drops at 10 weeks) | \|  \|  \| / \|  \|  \| / \| 2 \| 0 \|  \|  \| \| --- \| --- \| --- \| --- \| --- \| --- \| --- \| --- \| --- \| --- \| \| D \| D \|  \| M \| M \|  \| Y \| Y \| Y \| Y \| |  |
| at 10 weeks of age | | | |
| IM0508 | Penta-2 (Pentavalent 2 at 10 weeks) | \|  \|  \| / \|  \|  \| / \| 2 \| 0 \|  \|  \| \| --- \| --- \| --- \| --- \| --- \| --- \| --- \| --- \| --- \| --- \| \| D \| D \|  \| M \| M \|  \| Y \| Y \| Y \| Y \| |  |
| IM0509 | PCV-2 (Pneumococcal Conjugate at 10 weeks) | \|  \|  \| / \|  \|  \| / \| 2 \| 0 \|  \|  \| \| --- \| --- \| --- \| --- \| --- \| --- \| --- \| --- \| --- \| --- \| \| D \| D \|  \| M \| M \|  \| Y \| Y \| Y \| Y \| |  |
| IM0510 | RV-2 (Rotavirus vaccine at 10 weeks) | \|  \|  \| / \|  \|  \| / \| 2 \| 0 \|  \|  \| \| --- \| --- \| --- \| --- \| --- \| --- \| --- \| --- \| --- \| --- \| \| D \| D \|  \| M \| M \|  \| Y \| Y \| Y \| Y \| |  |
| at 14 weeks of age | | | |
| IM0511 | OPV-3 (Polio drops at 14 weeks) | \|  \|  \| / \|  \|  \| / \| 2 \| 0 \|  \|  \| \| --- \| --- \| --- \| --- \| --- \| --- \| --- \| --- \| --- \| --- \| \| D \| D \|  \| M \| M \|  \| Y \| Y \| Y \| Y \| |  |
| IM0512 | Penta-3 (Pentavalent 1 at 14 weeks) | \|  \|  \| / \|  \|  \| / \| 2 \| 0 \|  \|  \| \| --- \| --- \| --- \| --- \| --- \| --- \| --- \| --- \| --- \| --- \| \| D \| D \|  \| M \| M \|  \| Y \| Y \| Y \| Y \| |  |
| IM0513 | PCV-3 (Pneumococcal Conjugate at 14 weeks) | \|  \|  \| / \|  \|  \| / \| 2 \| 0 \|  \|  \| \| --- \| --- \| --- \| --- \| --- \| --- \| --- \| --- \| --- \| --- \| \| D \| D \|  \| M \| M \|  \| Y \| Y \| Y \| Y \| |  |
| IM0514 | IPV (INJECTABLE POLIO VACCINE AT 14 WEEKS) | \|  \|  \| / \|  \|  \| / \| 2 \| 0 \|  \|  \| \| --- \| --- \| --- \| --- \| --- \| --- \| --- \| --- \| --- \| --- \| \| D \| D \|  \| M \| M \|  \| Y \| Y \| Y \| Y \| |  |
| at 9 Months of age | | | |
| IM0515 | Measles-1 (AT 9 months) | \|  \|  \| / \|  \|  \| / \| 2 \| 0 \|  \|  \| \| --- \| --- \| --- \| --- \| --- \| --- \| --- \| --- \| --- \| --- \| \| D \| D \|  \| M \| M \|  \| Y \| Y \| Y \| Y \| |  |
| IM0515A | Typhoid TCV (at 9 months) | \|  \|  \| / \|  \|  \| / \| 2 \| 0 \|  \|  \| \| --- \| --- \| --- \| --- \| --- \| --- \| --- \| --- \| --- \| --- \| \| D \| D \|  \| M \| M \|  \| Y \| Y \| Y \| Y \| |  |
| IM0515B | IPV2 (at 9 months) | \|  \|  \| / \|  \|  \| / \| 2 \| 0 \|  \|  \| \| --- \| --- \| --- \| --- \| --- \| --- \| --- \| --- \| --- \| --- \| \| D \| D \|  \| M \| M \|  \| Y \| Y \| Y \| Y \| |  |
| IM0516 | Measles-2 (AT 15 months) | \|  \|  \| / \|  \|  \| / \| 2 \| 0 \|  \|  \| \| --- \| --- \| --- \| --- \| --- \| --- \| --- \| --- \| --- \| --- \| \| D \| D \|  \| M \| M \|  \| Y \| Y \| Y \| Y \| |  |
| IM07 | Check IM0501-IM0516: Are all vaccines (BCG to Measles-2) recorded? | Yes 1  No 2 | If 1 skip to IM22A  If 2 go back to IM05_1 |
|  | **INSTRUCTION**  *If IM07 = 1 go to IM22A*  *If IM07 = 2, please fill all vaccines above in IM05* | |  |
| IM08 | Has (name) ever received any vaccinations to prevent (him/her) from getting diseases, including vaccinations received in a campaign, immunization day or Child Health Day? | Yes 1  No 2  Don’t know 98 | If 2 or 98 skip to IM24 |
| IM09 | Has (name) ever received a BCG vaccination against tuberculosis – that is, an injection in the arm or shoulder that usually causes a scar? | Yes 1  No 2  Don’t know 98 |  |
| IM10 | Has (*name*) ever received any vaccination drops in the mouth to protect (him/her) from polio?  Probe by indicating that the first drop is usually given at birth and later at the same time as injections to prevent other diseases. | Yes 1  No 2  Don’t know 98 | If 2 or 98 GO TO IM11A |
| IM12 | How many times was the polio vaccine received at Govt/Public health facility or from outreach facility? | Number of times __ __  Don’t know 98 |  |
| IM11 | Were the first polio drops received in the first two weeks after birth? | Yes 1  No 2  Don’t know 98 |  |
| IM11A | Has (name) ever received Hepatitis B vaccination- that is, an injection in the arm or shoulder.  Hepatitis B vaccine protects against a viral infection that causes inflammation and damage to the liver۔ | Yes 1  No 2  Don’t know 98 |  |
| IM14 | Has (name) ever received a pentavalent vaccination – that is, an injection in the thigh to prevent (him/her) from getting tetanus, whooping cough, diphtheria, hepatitis b disease, and haemophilus influenza type b?  Probe by indicating that pentavalent vaccination is sometimes given at the same time as the polio drops. | Yes 1  No 2  Don’t know 98 | If 2 skip to IM16  If 98 skip to IM16 |
| IM15 | How many times was the pentavalent vaccine received? | Number of times __ __  Don’t know 98 |  |
| IM16 | Has (name) ever received a pneumococcal conjugate vaccination – that is, an injection to prevent (him/her) from getting pneumococcal disease, including ear infections and meningitis caused by pneumococcus?  Probe by indicating that pneumococcal conjugate vaccination is sometimes given at the same time as the pentavalent vaccination | Yes 1  No 2  Don’t know 98 | If 2 skip to IM18  If 98 skip to IM18 |
| IM17 | How many times was the pneumococcal vaccine received? | Number of times __ __  Don’t know 98 |  |
| IM18 | Has (name) ever received drops for rotavirus vaccine (RV) vaccination- a vaccine given orally at 6 & 10 weeks of age to protect against rotavirus infections, which are the leading cause of severe diarrhoea among young children? | Yes 1  No 2  Don’t know 98 | If 2 skip to IM20  If 98 skip to IM20 |
| IM19 | How many times were the drops received? | Number of times  Don’t know 98 |  |
| IM20 | Has (name) ever received an inactivated polio vaccine (IPV) – that is, a shot in the thigh at the age of 14 weeks and at 9 months to prevent children from getting polio? | Yes 1  No 2  Don’t know 98 | If 2 skip to IM21  If 98 skip to IM21 |
| IM20A | How many times was a IPV injection vaccine received? | Number of times  Don’t know 98 |  |
| IM21 | Has (name) ever received a measles injection – that is, a shot in the arm at the age of 9 months and 15 months of age to prevent children from getting measles? | Yes 1  No 2  Don’t know 98 | IF 2 OR 98 SKIP TO IM25A |
| IM22 | How many times was a measles injection vaccine received? | Number of times __ __  Don’t know 98 |  |
| IM22A | Has (name) ever received a Typhoid vaccination – that is, a shot in the upper part of the arm at the age of 9 months or older - to prevent (him/her) from getting Typhoid disease? | Yes 1  No 2  Don’t know 98 |  |
| IM22A | Has the child ever received polio drops during a national polo campaign? | Yes 1  No 2  Don’t know 98 | If no or DK, skip to IM23 |
| IM22B | How many times has (name) received polio drops during the national polio campaigns? |  |  |
| IM22C | Did (name) receive polio drops during the last campaign | Yes 1  No 2  Don’t know 98 | If 2 or 98 skip to IM22E |
| IM22D | The last time (name) received the polio drops, did (he/she) also get an injection to protect against polio?  Probe to ensure that both were given, drops and injection | Yes 1  No 2  Don’t know 98 |  |
| IM22E | Why did (name) not receive polio drops during the last campaign?  Record all the reasons mentioned but do not prompt by asking specific. Encourage the mother to provide all reasons. | Not at home when vaccinator came 1  Time of immunization not convenient 2  Family problem 3  Child was ill 4  Long wait……………………………………………………….5  Took child but no vaccine available 6  Took child but no vaccinator………………………….7  COVID-19-related (fear of going out, sickness in family) ……………………………………………………………8  No faith in immunization 9  Fear of reaction…………………………………………….10  Repeated campaigns……………………………………11  Contents of vaccine not halal………………………12  Vaccine leads to infertility…………………………13  Don’t know 98  Others 96  Please Specify___________________________ | Skip if IM22C = 1 |
| IM23 | Where is (name) usually vaccinated?  If the child is vaccinated from multiple sources, mention the usual source with higher frequency of vaccine doses. | Govt. Health facility 1  Private health facility 2  Government outreach service 3  Naunehalhealth camps…………………………………4  Others 96  Please Specify___________________________ | If IM23 = 3, skip to IM25 |
| IM23A | What means of transportation do you use for visiting the facility for immunization of (name)? | By foot 1  Cycle 2  Motorcycle/Rickshaw 3  Car / Taxi 4  Public Transport 5  Others 96  Please Specify __________________________ |  |
| IM23B | What is the time taken to reach this facility where vaccination was received? | Hours: Minutes |  |
| IM24 | Why (name) is not vaccinated?  If the child has not received all their vaccinations, ask the mother/caretaker.  Record all the reasons mentioned but do not prompt by asking specific. Encourage the mother to provide all reasons. | Place of immunization too far 1  Time of immunization not convenient 2  Mother too busy 3  Family problem including mother ill 4  Child ill, not brought 5  Child ill, brought but not vaccinated 6  Long wait 7  Rumors 8  No faith in immunization 9  Fear of side reaction 10  Time or place of immunization not known 11  Took child but no vaccine available 12  Took child but no vaccinator 13  Took child facility closed 14  Child was sick 15  Took child but not vaccination day 16  COVID-19-related (fear of going out, sickness in family) 17  Don’t know 98  Others 96  Please Specify___________________________ |  |
| IM25 | Observe BCG scar | Scar present 1  Scar absent 2  Child not available 3  Not observed 4 |  |

**Section PD: Care seeking during pregnancy and delivery**

**(To be administered to the mother of the youngest under-five child in the household)**

| Sr. No. | **Questions** | **Responses** | **Skip** |
| --- | --- | --- | --- |
| PD01 | Child’s Line Number (youngest under 5) |  |  |
| PD02 | WRA’s Name |  |  |
| PD03 | When was your last pregnancy? | 0-5.9 months back 1  6-11.9 months back 2  12-23.9 months back 3  24 months back or longer 4 |  |
| PD04 | Did you seek antenatal care during your last pregnancy? | Yes 1  No 2  Don’t know 98 | If no, skip to PD08 |
| PD05 | From whom did you seek antenatal care? | **HEALTH PERSONNEL**  Doctor 1  Nurse 2  Midwife 3  LHV 4  **OTHER PERSON**  Dai-TBA 5  Lady Health Worker 6  Homeopath 7  Hakim 8  Dispenser/Compounder 9  Others 96  Please Specify___________________________ |  |
| PD06 | Where did you seek antenatal care? | **Home**  Respondent’s home 1  Other home 2  **Public medical sector**  Government hospital 3  Government clinic 4  Mother & child health centre 5  Other public 961  Please Specify________________________  **Private medical sector**  Private hospital 7  Private clinic 8  Private maternity home 9  Naunehalhealth camp…………………………………10  other private 962  Please Specify____________________ |  |
| PD07 | How many times did you receive antenatal care during last pregnancy?  Probe to identify the number of times antenatal care was received. If a range is given, record the minimum number of times antenatal care received. | Number of times [__] [__]  Don’t Know 98 |  |
| PD08A | As part of your antenatal care during your pregnancy, were any of the following done at least once:  (ASK ABOUT EACH OPTION AND CIRCLE ALL POSITIVE RESPONSES) | Were you weighed ------------------------------------1  Was your BP measured -------------------------------2  Did you give urine sample ----------------------------3  Did you give blood sample ---------------------------4  Ultrasound -----------------------------------------------5  Received counselling on nutrition ------------------6  Received counselling on breastfeeding -----------7  Received counselling on family planning ---------8  none of the above --------------------------------------9  *Others ............................................................ 96*  **Please Specify___________________________** |  |
| PD08 | Why did you not seek antenatal care? | Fear of COVID-19 1  Assumption that care is not needed during pregnancy 2  On the advice of a relative or friend 3  Family knew how to care for you themselves 4  Financial reasons 5  Limitations of transport 6 |  |
| PD08B | During your last pregnancy, did you receive any tetanus injections | Yes ................................................................... 1  No ...................................................................2  Don’t *know* ................................................... 98 | If no skip to PD09 |
| PD08C | How many times did you receive tetanus injection? | *___ _____* No. of Times |  |
| PD09 | Have you ever taken any iron folic acid (IFA) while you were pregnant? | Yes 1  No 2 | If no skip to PD12 |
| PD10 | How often did you take IFA during pregnancy? | Daily 1  Once a week 2  Biweekly 3  Monthly 4  Rarely 5 |  |
| PD11 | How many months or days did you take IFA? | Months [__] [__]  Days [__] [__] |  |
| PD12 | Who conducted the delivery? | **HEALTH PERSONNEL**  Doctor 1  Nurse 2  Midwife 3  LHV 4  CMW 5  **OTHER PERSON**  Dai-TBA 6  Lady Health Worker 7  Others 96  Please Specify___________________________ |  |
| PD13 | During you last birth, where was the baby born? | Own home 1  Other home 2  Government Hospital 3  RHC 4  BHU 5  birth station 6  Private Hospital/Clinic 7  enroute to facility 8  Other Public Facility 961  Please Specify_________________________  Other Private Facility 962  Specify________________________________ | If not 1 or 2 then skip to PD15 |
| PD14 | If at home, why was the delivery not in a facility? | Fear of COVID-19 1  All my deliveries have been at home 2  My family did not want me to go to  a facility 3  Financial reasons 4  Limitations of transport 5  Health Facility is far from home 6  Others 96  Please Specify___________________________ |  |
| PD15 | Did anyone check on your health after delivery? | Yes 1  No 2 | If no, DK skip to PD19 |
| PD16 | Who checked your health after delivery?  [Multiple responses] | Gynaecologist 1  Other doctor 2  Nurse 3  CMW 4  LHW 5  LHV 6  TBA 7  Others 96  Please Specify___________________________ |  |
| PD17 | How long after delivery did the first of these checks happen?  If less than one day, record hours.  If less than one week, record days.  Otherwise, record weeks. | Hours [__][__] 01  Days [__][__] 02  Weeks [__][__] 03  Don’t know/ don’t remember 98 |  |
| PD18 | How many times such check-ups happen? | Times [__][__] |  |
| PD19 | Did anyone check on (name of child) health? | Yes 1  No 2  Don’t know 98 | If no or DK skip to PD23 |

| PD20 | Who checked the child’s health after delivery?  [Multiple responses] | Paediatrician 1  Other doctor 2  Nurse 3  CMW 4  LHW 5  LHV 6  TBA 7  Others 96  Please Specify___________________________ |  |
| --- | --- | --- | --- |
| PD21 | How long after delivery did the first of these check-ups Conducted?  If less than one day, record hours.  If less than one week, record days.  Otherwise, record weeks. | Hours [__][__] 01  Days [__][__] 02  Weeks [__][__] 03  Don’t know/ don’t remember 98 |  |
| PD22 | How many times such check-ups have been Conducted? | Times [__][__] |  |
| PD23 | After the cord was cut and until it fell off, was anything applied to the cord? | Yes ................................................................... 1  No .................................................................... 2  Don’t know 98 |  |
| PD24 | What was applied to the cord?  *[MULTIPLE RESPONSES]* | Chlorhexidine .................................................. 1  Other antiseptic (alcohol, spirit, gentian violet, Dettol) ............................................................. 2  Surma ..............................................................3  Mustard oil ..................................................... 4  Ash ................................................................... 5  Animal dung .................................................... 6  Others ............................................................ 96  Please Specify___________________________  Don’t know / don’t remember 98 | skip if PD23 is no or DK |

**Section BF: Breastfeeding, Health & Care-seeking Behaviors**

Instruction: To be administered to the mother of the youngest child in the household, between 0-23 months of age

| **Sr. No.** | **Questions** | **Responses** | **Skip** |
| --- | --- | --- | --- |
| BF01 | Child’s Line Number (youngest under 2) |  |  |
| BF02 | WRA’s Name |  |  |
| BF03 | What is (NAME) birthday? | Year _____ Month ________ Date_______ |  |
| BF03A | Child’s age | Months _____  Years _______ |  |
| BF04 | Has (NAME) ever been breastfed? | Yes 1  No 2  Don’t know 98 | If no, or don’t know skip to BF11 |
| BF05 | How long after birth did you first put (NAME) to the breast? | 1. Immediately___ OR 2. Hours _____ OR 3. Days _____ OR   66. Never _____ |  |
| BF06 | Did you feed first milk (colostrum) to the child? | Yes 1  No 2  Don’t know 98 | If yes, skip to BF08 |
| BF07 | Why did you not feed colostrum to (NAME)? | Bad milk 1  Harmful for child 2  Thickness 3  Others 96  Please Specify___________________________ |  |
| BF08 | Was (NAME) given anything to drink before breast milk? | Yes 1  No 2  Don’t know 98 | If no or don’t know then skip to BF10 |
| BF09 | What was (NAME) given to drink? | Milk (other than breast milk) 1  Plain water 2  Sugar or glucose water 3  Gripe water 4  Sugar-salt-water solution 5  Fruit juice 6  Infant formula 7  Tea / Infusions / Traditional herbal preparations 8  Honey 9  Prescribed medicine 10  Others 96  Please Specify___________________________  Not given anything to drink 99 |  |
| BF10 | Was (NAME) breastfed yesterday during the day or at night? | Yes 1  No 2  Don’t know 98 | If yes, skip to BF12 |
| BF11 | Sometimes babies are fed breast milk in different ways, for example by spoon, cup, or bottle. This can happen when the mother cannot always be with her baby. Sometimes babies are breastfed by another woman, or given breast milk from another woman by spoon, cup or bottle or some other way. This can happen if a mother cannot breastfeed her own baby.  Did (NAME) consume breast milk in any of these ways yesterday during the day or at night? | Yes 1  No 2  Don’t know 98 |  |
| BF12 | Now I would like to ask you about some medicines and vitamins that are sometimes given to infants.  Was (NAME) given any vitamin drops or other medicines as drops yesterday during the day or at night? | Yes 1  No 2  Don’t know 98 |  |
| BF13 | Was (NAME) given ORS/NIMKOL yesterday during the day or at night? | Yes 1  No 2  Don’t know 98 |  |
| BF14 | Next I would like to ask you about some liquids that (NAME) may have had yesterday during the day or at night.  Did (NAME) have any (ITEM FORM LIST)?  READ THE LIST OF LIQUIDS STARTING WITH ‘PLAN WATER’ | Plain water ............................................. Y/N/DK  Infant formula ........................................ Y/N/DK  Infant formula ............................... Times __  Milk such as tinned, powdered, or fresh animal milk ......................................................... Y/N/DK  Milk such as tinned, powdered, or fresh animal milk ............................................... . Times __  Juice or juice drinks ............................... Y/N/DK  Clear broth.............................................. Y/N/DK  Yogurt ..................................................... Y/N/DK  Yogurt ............................................ Times __  Thin porridge .......................................... Y/N/DK  Any other liquids such as [list other water-based liquids available in the local setting] ...... Y/N/DK  Any other liquids .................................... Y/N/DK |  |
| BF15 | Please describe everything that (NAME) ate yesterday during the day or night, whether at home or outside the home. | Porridge, bread, rice, noodles, or other foods made from grains ................................... Y/N/DK  Pumpkin, carrots, squash, or sweet potatoes that are yellow or orange inside ............. Y/N/DK  White potatoes, white yams manioc, cassava, or any other food made from roots ............ Y/N/DK  Any dark green leafy vegetables ............ Y/N/DK  Ripe mangoes, ripe papayas ................... Y/N/DK  Any other fruits or vegetables ................ Y/N/DK  Liver, kidney, heart, or other organ meats ................................................................ Y/N/DK  Any meat, such as beef, lamb, goat chicken, or duck ........................................................ Y/N/DK  Eggs ........................................................ Y/N/DK  Fresh or dried fish, shellfish, or seafood Y/N/DK  Any food made from beans, peas lentils, nuts, or seeds .................................................. Y/N/DK  Cheese, yogurt, or other milk product ... Y/N/DK  Any oil, fats, or butter, or foods made with any of these................................................... Y/N/DK  Any sugary foods such as chocolates, sweets candies, pastries cakes, or biscuits ......... Y/N/DK  Condiments for flavor, such as chilies, spices, herbs, or fish powder ............................. Y/N/DK  Foods made with red palm oil, red palm nut, or red palm nut pulp sauce ......................... Y/N/DK |  |
|  | Check responses in BF15, if all ‘no’ for BF15, go BF16. If at least one ‘yes’ or all ‘DK’ go to BF17 | | |
| BF16 | Did (NAME) eat any solid, semi- solid, or soft foods yesterday during the day or at night?  IF ‘YES’ PROBE: what kind of solid, semi-solid, or soft foods did (NAME) eat?  If BF16 is “yes” & all responses to BF15 are “no” please go back to BF15 and record foods items consumed. Then continue with BF17. | Yes ................................................................... 1  No .................................................................... 2  Don’t know ................................................... 98 | If no or DK, skip to BF18 |
| BF17 | How many times did (NAME) eat solid, semi-solid, or soft foods other than liquids yesterday during the day or at night? | Number of times ------------------------------ [__][__]  Don’t know ................................................... 98 |  |
| BF18 | Did (NAME) drink anything from a bottle with a nipple yesterday during the day or night? | Yes ................................................................... 1  No .................................................................... 2  Don’t know ................................................... 98 | If no, skip to BF20 |
| BF19 | What did (name) drink from the bottle with a nipple? | Top feed (formula milk) ------------------------------ 1  Animal milk --------------------------------------------- 2  Expressed milk ---------------------------------------- 3  Others ............................................................ 96  Please Specify ___________________________ |  |
| BF20 | Did (name) drink or eat vitamin or mineral supplements or any medicines yesterday, during the day or night? | Top feed (formula milk) ------------------------------ 1  Animal milk --------------------------------------------- 2  Expressed milk ---------------------------------------- 3  Others ............................................................ 96  Please Specify ___________________________ |  |

**Section G5: COVID (To be administered to the mother of the youngest under-five child in the household)**

| **Sr. No.** | **Questions** | **Responses** | **Skip** |
| --- | --- | --- | --- |
| CV01 | Have you heard of COVID-19? | Yes 1  No 2  Don’t know 98 | If no, skip to section SE |
| CV02 | How concerned are you about COVID-19? | Not Worried 1  A Little Worried 2  Somewhat Worried 3  Very Worried 4  Extremely Worried 5 |  |
| CV03 | Do you think COVID-19 exists? | Yes, it exists 1  No, it doesn’t 2  Don’t know 3 |  |
| CV04 | How deadly do you think COVID-19 is? | Not At All Deadly 1  Not Very Deadly 2  Not Deadly 3  Somewhat Deadly 4  Extremely Deadly 5 |  |
| CV05 | Do you know which group of people are most at risk of COVID? | Everyone 1  Elderly 2  Adults 3  Children 4  Pregnant/Lactating Women 5  Health Workers 6  Person With Pre-Existing Conditions 7  Others 96  Please Specify___________________________ |  |
| CV06 | Who do you trust for information around COVID?  (Choose all that apply. Do not read the answers, let the respondent answer spontaneously) | Community Leader 1  Religious Leader 2  Relatives 3  Friends 4  Health Worker At Health Facility 5  Health Worker Via Door To Door 6 Campaign 7  Newspaper 8  Radio 9  Television 10  Social Media 11  NaunehalHealth Camps………………………………12  Others 96  Please Specify___________________________ |  |
| CV07 | Will all people with COVID-19 show symptoms? | Yes 1  No 2  Don’t know 98 |  |
| CV08 | What symptoms are most commonly reported with COVID-19 (check all that apply) | Fever 1  Cough 2  Flu 3  Sore throat 4  Chest congestion/Difficulty in breathing 5  Losing sense of smell & taste 6  Diarrhea 7  Don’t know 98  Others 96  Please Specify___________________________ |  |
| CV09 | What do you think are the different modes of transmission of COVID-19?  (Choose all that apply. Do not read the answers, let the respondent answer spontaneously) | Touching surfaces contaminated with the virus 1  Shaking hands 2  Directly coming in contact with an infected person’s body fluids such as blood, vomit, or sweat 3  Via droplets entering through mouth or nose when an infected person sneezes or coughs 4  Unhygienic food preparation 5  Radio waves such as mobile networks 6  Mosquito bite 7  COVID vaccination………………………………………….8  Don’t know 98  Others 96  Please Specify___________________________ |  |
| CV10 | What according to you are effective methods of preventing infection from COVID-19?  (Choose all that apply. Do not read the answers, let the respondent answer spontaneously) | Frequently washing hands using soap and water for 20 seconds 1  Cleaning hands with hand sanitizer 2  Covering mouth and nose using a tissue or flexed elbow while coughing or sneezing 3  Maintain social distancing 4  Avoiding contact with sick people showing signs of COVID-19 5  Wearing a mask in public places 6  Keep oneself isolated 7  Praying to God 8  COVID vaccination…………………………………………9  Don’t know 98  Others 96  Please Specify___________________________ |  |
| CV11 | Has COVID-19, the subsequent lockdown & restrictions and fear affected the nutrition and feeding of your child? | Yes 1  No 2 | If no, skip to CV13 |
| CV12 | How has it affected breastfeeding and/or feeding of the child?  (Choose all that apply. Do not read the answers, let the respondent answer spontaneously) | I have reduced the frequency and amount I feed my child 1  I have increased the frequency and amount I feed my child 2  I cannot afford same food for child 3  I cannot travel to markets to purchase food 4  The foods I feed my child are no longer available in the market 5  Others 96  Please Specify___________________________ |  |
| CV13 | Has COVID-19, the subsequent lockdown & restrictions and fear disrupted your child’s immunization? | Yes 1  No 2 |  |
| CV14 | Has your child (under-5) received oral polio vaccine in the past 12 months? | Yes 1  No 2 |  |
| CV15 | Has your child received all vaccinations in the past 12 months that were due as per EPI schedule? | Yes 1  No 2 |  |
| CV16 | If not, why was the child not taken for vaccination? | Fear of COVID-19 1  Financial reasons 2  Limitations of transport 3  Vaccinations can be delayed 4  Forgot that there was a vaccine due 5  Interrupted services 6  Others 96  Please Specify___________________________ |  |
| CV17 | Has the number of times you visit health facilities reduced during COVID-19? | Yes 1  No 2 | If no, skip to CV19 |
| CV18 | Why has it decreased? | Fear of COVID-19 1  Financial reasons 2  Limitations of transport 3  Vaccinations can be delayed 4  Forgot that there was a vaccine due 5  Interrupted services 6  Others 96  Please Specify___________________________ |  |
| CV19 | For what conditions have you decided to not go to the doctor? | My child had diarrhea 1  My child had fever or cough 2  My/my wife’s pregnancy and/or childbirth 3  My child’s health issue, prefer not specify 4  My health, prefer not to specify 5  Other, prefer not to say 6  Others 96  Please Specify___________________________ |  |
| CV 20 | Have you received COVID vaccination? | Yes 1  No 2 | If Yes, go directly to next section.  If No, go to next question |
| CV 21 | Why did you not get the COVID vaccine?  (Choose all that apply. Do not read the answers, let the respondent answer spontaneously) | Place of immunization too far 1  Time of immunization not convenient 2  Vaccine was not available at facility………………3  Time or place of immunization not known ..4  Family problem 5  Long wait 6  No faith or trust in COVID vaccine 7  Fear of side effects ..8  COVID-19-related (fear of going out, sickness in family) 9  Don’t know 98  Others 96  Please Specify___________________________ |  |

**Section SE: Household Characteristics**

| **Sr. No.** | **Questions** | **Responses** | **Skip** |
| --- | --- | --- | --- |
| SE01 | What is the mother tongue of the head of the household? | Urdu 1  Punjabi 2  Sindhi 3  Pushtu 4  Baluchi 5  Siraiki 6  Hindko 7  Others 96  Please Specify___________________________ |  |
| SE02 | Main material of the dwelling floor | **Natural Floor**  Mud/Clay/Earth/Sand 1  Dung 2  **Rudimentary/Floor**  Wood planks 3  Palm/Bamboo 4  **Finished Floor**  Parquet or polished wood 5  Vinyl or asphalt strips 6  Ceramic tiles 7  Cement 8  Carpet 9  Bricks 10  Lime 11  Others 96  Please Specify___________________________ |  |
| SE03 | What is the primary construction material used to build the house (by observation)? | **NATURAL WALLS**  No Walls 1  Cane/Palm/Trunks 2 Dirt 3  Mud/Stones 4  Bamboo/Sticks/Mud 5  **RUDIMENTARY WALLS**  Unbaked Bricks/Mud 6  Bamboo With Mud 7  Stone With Mud 8  Uncovered Adobe 9  Plywood 10  Reused Wood 11  **FINISHED WALLS**  Cement 12  Stone With Lime/Cement 13  Bricks 14  Cement Blocks 15  Covered Adobe 16  Wood Planks/Shingles 17  Others 96  Please Specify___________________________ |  |
| SE04 | Observe the primary construction material  Used to build the roof? | **NATURAL ROOFING**  No Roof 1  Thatch/Palm Leaf 2  Sod/Grass 3  **RUDIMENTARY ROOFING**  Rustic Mat 4  Palm/Bamboo 5  Wood Planks 6  Cardboard 7  **FINISHED ROOFING**  Asbestos 8  Reinforced Brick Cement/R 9  Metal 10  Wood 11  Calamine/Cement Fiber 12  Ceramic Tiles 13  Cement/RCC 14  Roofing Shingles 15  Others 96  Please Specify ______________________ |  |
| SE05 | What is the ownership status of this house? | Owned 1  Rented 2  Living without paying rent 3  Others 96  Please Specify___________________________ | If the  Answer  Is 1,3 OR  96, skip  to SE07 |
| SE06 | If this house is rental, what is the monthly rent of this house? | Rupees: ______________________________ |  |
| SE07 | How many rooms in this HH are used for sleeping? | number: |  |
| SE08 | What is the main source of drinking water  In this HH? | **PIPED WATER**  Piped Into Dwelling 1  Piped To Yard/Plot 2  Piped To Neighbor 3  Public Tap/Standpipe 4  Tube Well Or Borehole 5  **DUG WELL**  Protected Well 6  Unprotected Well 7  **WATER FROM SPRING**  Protected Spring 8  Unprotected Spring 9  Rainwater 10  Tanker Truck 11  Cart With Small Tank 12  Surface Water (River/Dam/  Lake/Pond/Stream/Canal/  Irrigation Channel) 13  Bottled Water 14  Others 96  Please Specify___________________________ |  |
| SE09 | Do you do anything to the water to make it safer to drink? | Yes 1  No 2  Don’t know 98 | If no, skip to SE11 |
| SE10 | What do you usually do to make the water safer to drink?  Anything else?  RECORD ALL MENTIONED. | Boil 1  Add Bleach/Chlorine 2  Strain Through A Cloth 3  Use Water Filter (Ceramic/  Sand/Composite/Etc) 4  Solar Disinfection 5  Let It Stand And Settle 6  Other 96  Pleas Specify___________________________  Don't Know 98 |  |
| SE11 | What kind of toilet facility do members of your HH usually use? | **FLUSH OR POUR FLUSH TOILET**  Flush To Piped Sewer System 1  Flush To Septic Tank 2  Flush To Pit Latrine 3  Flush To Somewhere Else 4  Flush, Don't Know Where 5  **PIT LATRINE**  Ventilated Improved Pit Latrine 6  Pit Latrine With Slab 7  Pit Latrine Without Slab/Open Pit 8  Composting Toilet 9  Bucket Toilet 10  Hanging Toilet/Hanging Latrine 11  No Facility/Bush/Field 12  Others 96  Please Specify___________________________ |  |
| SE12 | What is the drainage system of this HH?  (Through observation) | Underground sewerage lines 1  Open sewerage line 2  Unattached sewerage lines 3  Others 96  Please Specify___________________________ |  |
| SE13 | We would like to learn about the places that households use to wash their hands. Can you please show me where members of your household most often wash their hands? | Observed, Fixed Place 1  Observed, Mobile 2  **NOT OBSERVED**  Not in Dwelling/Yard/Plot 3  Not Observed, No Permission to See 4  Not Observed, Other Reason 5 | If not observed,  then skip to SE16 |
| SE014 | Observe presence of water at the place of washing  *RECORD OBSERVATION* | Water Is Available 1  Water Is Not Available 2 |  |
| SE15 | Observe presence of soap, detergent, or other cleansing agent at the place for handwashing.  *RECORD OBSERVATION* | Soap or Detergent (Bar, Liquid, Powder, Paste) 1  Ash, Mud, Sand 2  None 3 |  |
| SE16 | Do you have any soap or detergent in your household for hand washing? | Yes 1  No 2 | If no, skip to SE18 |
| SE17 | If yes, can you please show it to me?  [Multiple responses] | Bar soap 1  Detergent (powder/liquid/paste) 2  Liquid soap 3  Ash/mud/sand 4  None 5 |  |
| SE18 | In what situations do you wash your hands with soap?  *This is an open-ended question. Do not read the answer choices.* | 1. Before preparing food Y / N 2. Before eating Y / N 3. Before feeding a child Y / N 4. After handling feces/diapers Y / N 5. After defecating or using the latrine Y / N   96. Others Y / N  Please Specify ______________________ |  |
| SE19 | What fuel does your HH use for cooking? | Electricity 1  LPG 2  Natural Gas 3  Biogas 4  Kerosene 5  Coal, Lignite 6  Charcoal 7  Wood 8  Straw/Shrubs/Grass 9  Agricultural Crop 10  Animal Dung 11  No Food Cooked In Household 12  Others 96  Please Specify ______________________ |  |
| SE20 | What transport does most of the family members  of this HH usually use? (select all that apply) | Private car 1  Company car 2  Motorcycle Public transport 3  By walk 4  Cycle 5  Others 96  Please Specify ______________________ |  |
| SE21 | What technique do you use to stop the breeding of flies and mosquitos?  [Multiple responses] | Spray 1  Mat 2  Mosquito net 3  Globe 4  Smoke Or frankincense 5  Others 96  Please Specify ______________________ |  |
| SE22 | Does your household have | Electricity Y/N  Radio Y/N  Television Y/N  Non-Mobile Telephone Y/N  Refrigerator Y/N  Almirah/Cabinet Y/N  Chair Y/N  Room Cooler Y/N  Air conditioner Y/N  Washing Machine Y/N  Water Pump Y/N  Bed Y/N  Clock Y/N  Sofa Y/N  Camera Y/N  Sewing Machine Y/N  Computer Y/N  Internet Connection Y/N |  |
| SE23 | Does anyone in your household own a mobile phone? | Yes 1  No 2 | If no, then  Skip to SE31 |
| SE24 | How many mobile phones are there in your household? |  |  |
| SE25 | How many of these are smartphones? |  | If none, then skip  To SE31 |
| SE26 | How many of these smartphones are Android-based? |  |  |
| SE27 | Which mobile service provider is the Android-based smartphone connected to?  *[Multiple responses]* | Ufone 1  Jazz 2  Zong 3  Telenor 4 |  |
| SE28 | What connectivity speed does the Android-based smartphone have?  *[Multiple responses]* | 2G 1  3G 2  4G 3 |  |
| SE29 | Does the caregiver of a young child in the household have access to the Android smartphone or not? | Yes 1  No 2 |  |
| SE30 | Who owns their own Android smart phone?  *[Multiple responses]* | Myself My mother-in-law/mother 1  My husband/wife My father-in-law/father 2  Children 3  Others 96  Please Specify___________________________ |  |
| SE31 | Where do your family members usually go to seek care? | Govt. Health care facility 1  Private health care facility 2  Others 96  Please Specify___________________________ |  |
| SE32 | From whom do your family members usually seek care? | **HEALTH PERSONNEL**  Doctor 1  Nurse 2  Midwife 3  LHV 4  **OTHER PERSON**  Dai-TBA 5  Lady Health Worker 6  Homeopath 7  Hakim 8  Dispenser/Compounder 9 |  |
| SE33 | How much time does it take to reach any govt or private health care facility near your house by walking? | Hours Minutes |  |
| SE51 | Are you aware of the *Naunehal*health camps? | Yes 1  No 2  Don’t know…………………………………………………..98 | If 2 or 98, then skip  all questions till  the current SE 48  about the health  promotion sessions |
| SE 41 | Have you or anyone from the household visited the *Naunehal*health camp? | Yes 1  No 2  Don’t know…………………………………………………..98 | If 1 SKIP TO SE43  If 2 skip ALL  QUESTION AND GOTO  SE48 |
| SE42 | Why did you or someone from the household not visit the *Naunehal*health camps?  (Choose all that apply. Do not read the answers, let the respondent answer spontaneously) | Did not know about them………………………………1  Did not need to go……………………………………….…2  No faith in immunization………………………………..3  Too far…………………………………………………………...4  Long wait………………………………………………………..5  Don’t know ..98  Others ..96  Please Specify___________________________ | If any response  Skip to 48 |
| SE43 | From where did you get information about the health camp? | Community Mobilizers of TVI/Prime Foundation…………………………………………..1  Community Mobilizers of UNICEF……..….2  FCVs…………………………………………………….3  Polio workers………………………………………4  LHWs………………………………………………….5  LHVs……………………………………………………6  Doctors……………………………………………….7  Nurses…………………………………………………8  Announcements at the mosque/ community…..9  Neighbors……………………………………………10  Friends and relatives…………………………11  Others (Specify) __________________96 |  |
| SE44 | For which specific purpose did you visit the health camp? | Seeking care for child…………………………1  Seeking care for mother……………………..2  Immunization of the child…………………..3  Unavailability of health services in the area….4  Others (Specify) ____________________96 |  |
| SE45 | Did you or your child receive any services at health camp? | Yes……………………………………………………….1  No …………………………………………….2  Do not know………………………………….98 | If 2 or Don’t know  Skip to SE47 |
|  |  |  |  |
| SE46 | Which services did you avail of at the *Naunehal*health camp?  (Choose all that apply. Do not read the answers, let the respondent answer spontaneously) | Child immunization…………………………………………1  Maternal tetanus immunization…………………….2  Consultation for child’s illness……………………….3  Consultation for adult illness………………………….4  Antenatal care………………………………………………..5  General checkup…………………………………………….6  Other……………………………………………………………96  Please specify: __________________________ |  |
| SE52 | Did you receive nutritional advice during your visit at the *Naunehal*health camp? | Yes……………………………………………………….1  No …………………………………………….2  Do not know………………………………….98 | If 2 or 98 then skip  next question and  go to SE 47 |
| SE53 | Which nutritional topic did you get advice on? | Breastfeeding counselling……………………………….1  Complementary feeding advice…………………….2  Nutritional advice on poor weight gain in child……………….3  Maternal nutrition………………………………..4  Other……………………………………………………………96  Please specify: __________________________ |  |
| SE54 | If child was taken, who accompanied the child to the camp?  (Choose all that apply) | Mother…………………………………………………………1  Father……………………………………………………………2  Grandfather…………………………………………………..3  Grandmother…………………………………………………4  Uncle/Aunt……………………………………………………5  Sibling…………………………………………………………….6  Other……………………………………………………………96  Please specify:__________________________ |  |
| SE47 | Mention reason why your child didn’t receive vaccination in the health camp? | Vaccines were not available at health camp……………………………………………………………...1  Camp staff/ physician did not recommend………2  Child was not well……………………………………………3  Child did not visit the health camp……………………………………………………………...4  Refused for vaccination…………………………………………….………5  Long waiting time……………………………………………………………….6  Vaccination already completed……………………..7  Others (Specify) _______________________96 | IF 4 SKIP TO SE48  Other wise goto SE55 |
| SE55 | Was there anything important that you think should have been added to mobile health services for mothers and under-five children? | Yes 1  Please specify…………………………………………………  No 2  Don’t know…………………………………………………..98 |  |
| SE 48 | Have you or anyone from household attended a *Naunehal*health promotion session? | Yes 1  No 2  Don’t know…………………………………………………..98 | If 2 skip to SE 50  If 98 skip to SE 50 |
| SE56 | Who attended a *Naunehal*health promotion session from your household?  (Multiple response) | Mother…………………………………………………………1  Father……………………………………………………………2  Grandfather…………………………………………………..3  Grandmother…………………………………………………4  Uncle/Aunt……………………………………………………5  Sibling…………………………………………………………….6  Other……………………………………………………………96  Please specify: __________________________ |  |
| SE 49 | Which topics were covered in the health promotion sessions?  (Choose all that apply. Do not read the answers, let the respondent answer spontaneously) | Immunization………………………………………………..1  WASH……………………………………………………………2  Child nutrition……………………………………………….3  Pregnancy and maternal health……………………4  Don’t know 98  Others 96  Please Specify___________________________ |  |
| SE 50 | Why did you or someone from the household not attend a health promotion session? | Did not know about them………………………………1  Did not feel the need to go ………………………….2  Too far…………………………………………………………...3  Don’t know 98  Others 96  Please Specify___________________________ |  |
| SE57 | Was there anything important that you think should have been covered in health promotion sessions for mothers and under-five children? | Yes 1  Please specify…………………………………………………  No 2  Don’t know…………………………………………………..98 |  |
| SE34 | For how long you have been living in this house? | Months: Years: |  |
| SE58 | Are you originally from this area? (This District) | Yes 1  No 2  Don’t know……………………………………………….98 | If Yes, or Don't Know  then skip next two  questions |
| SE59 | Where are you originally from? (if known enter city/ district, province, country) | -------------------------------------------------------------- |  |
| SE60 | Why did you move from there to your current location? | Work/Job……………………………………………………..1  Family/Personal reasons………………………………2  Educational reasons…………………………………….3  Health reasons…………………………………………….4  Violence or Conflict…………………………………….5  Natural disaster (drought, flood etc.)…………6  Don’t know 98  Others 96  Please Specify___________________________ |  |
| SE35 | Do you have any plans to shift somewhere else in near future? (In less than a year) | Yes 1  No 2  Don’t know 98 |  |
| SE36 | Has your household or anyone in your household received financial assistance in last 12 months? | Yes 1  No 2  Don’t know 98 | If no skip to SE40 |
| SE37 | If yes, then please identify which program they are registered with?  *[Multiple Responses]* | Unconditional Cash Transfer 1  Waseela-e-Haq 2  Waseela-e-Taleem 3  Waseela-e-Rozgar 4  Waseela-e-Sehat Bait ul Mal 5  EOBI 6  BISP 7  Workers Welfare Funds 8  National Income Support Program 9  Ehsas program……………………………………………10  Others 96  Please Specify___________________________ |  |
| SE38 | When was the last payment received from this program? | In the last one month 1  In the last 03 months 2  In the last 06 months 3  More than one year ago 4  More than two years ago 5  Don’t know 98 |  |
| SE39 | Is a financial assistance program receipt available?  Observed by surveyor.  RECORD OBSERVATION | Available and observed 1  Not observed 2 |  |
| SE40 | Have you or anyone in the household received a food supplement from an organization in the past 12 months? | Yes 1  No 2  Don’t know 98 |  |
